# A global systematic review and meta‐analysis on the babesiosis in dogs with special reference to *Babesia canis*

**DOI:** 10.1002/vms3.1427

**Published:** 2024-05-02

**Authors:** Amir Abdoli, Meysam Olfatifar, Milad Badri, Leila Zaki, Behzad Bijani, Majid Pirestani, Kareem Hatam‐Nahavandi, Aida Vafae Eslahi, Panagiotis Karanis

**Affiliations:** ^1^ Zoonoses Research Center Jahrom University of Medical Sciences Jahrom Iran; ^2^ Department of Parasitology and Mycology Jahrom University of Medical Sciences Jahrom Iran; ^3^ Gastroenterology and Hepatology Diseases Research Center Qom University of Medical Sciences Qom Iran; ^4^ Medical Microbiology Research Center Qazvin University of Medical Sciences Qazvin Iran; ^5^ Department of Parasitology and Entomology Faculty of Medical Sciences Tarbiat Modares University Tehran Iran; ^6^ Department of Parasitology and Mycology School of Medicine Iranshahr University of Medical Sciences Iranshahr Iran; ^7^ Medical Faculty and University Hospital University of Cologne Cologne Germany; ^8^ Department of Basic and Clinical Sciences University of Nicosia, Medical School, Anatomy Centre Nicosia Cyprus

**Keywords:** canine babesiosis, meta‐analysis, tick‐borne diseases, worldwide

## Abstract

**Background:**

Canine babesiosis is a clinically significant tick‐transmitted disease caused by several species of the intraerythrocytic protozoan parasite *Babesia*, which result in a wide range of clinical manifestations, from mild, transient infection to serious disease and even death.

**Objectives:**

The current study aimed to estimate the global prevalence and associated risk factors of *Babesia* in dogs.

**Methods:**

Multiple databases (PubMed, Scopus, ProQuest, Web of Science and Google Scholar) were searched for relevant literature published from January 2000 up to December 2022. The statistical analyses were performed based on the R software (version 3.6) meta‐package.

**Results:**

Out of 23,864 publications, 229 studies met the inclusion criteria. The pooled prevalence of canine babesiosis was 0.120 (95% CI; 0.097–0.146). The highest pooled prevalence was found in Europe (0.207, 95% CI; 0.097–0.344). Among several species, *Babesia canis* was the most prevalent parasite (0.216, 95% CI; 0.056–0.441). The highest pooled prevalence of *Babesia* in dogs was observed in the summer season (0.097, 95% CI; 0.040–0.174).

**Conclusions:**

Regular screening and appropriate control strategies are recommended for the prevention of transmission of tick‐borne disease transmission among dogs.

## Graphical abstract

Canine babesiosis is a clinically significant tick‐transmitted disease caused by several species of *Babesia* parasites. *Babesia canis* was the most prevalent parasite. Owned dogs had higher prevalence rate than stray/shelter dogs.

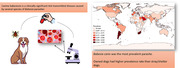

## 1 INTRODUCTION

Vector‐borne diseases (VBDs) are included among the emerging and re‐emerging infections, representing health concern for humans, livestock, wildlife, and companion animals (Kuleš et al., 2017). They cause significant economic losses due to high mortality rates and, as a result, decreases profit in the global livestock industry (Lew‐Tabor & Valle, 2016).

Several factors, including global development, urbanization, climate change, increased international trade, and animal travel and mobility, influence the epidemiology and distribution of VBDs (Baneth et al., 2012; Harrus & Baneth, 2005). Ticks are well‐known vectors for a broad range of microbial pathogens of both public health and veterinary importance (Bajer, Kowalec, et al., 2022; Efstratiou et al., 2021).

Babesiosis is a globally distributed tick‐borne disease caused by intra‐erythrocytic protozoa *Babesia* (Solano‐Gallego et al., 2016). In 1895, the disease was first observed in dogs in northern Italy (Penzhorn, 2020). The life cycle of *Babesia* parasites develops through the transmission of sporozoites from the salivary glands of ixodid ticks (the main vectors) to their vertebrate hosts, where the merozoites appear in red blood cells after the occurrence of asexual replication (merogony) (Antunes et al., 2017; Jalovecka et al., 2019; Vannier & Krause, 2020).

Dogs are considered the most frequent companion animals worldwide. The transmission of zoonotic pathogens from these animals to the human population is an inevitable concern (Dantas‐Torres et al., 2020; Eslahi et al., 2021; Omidinia et al., 2020). However, the canine *Babesia* species are assumed to have no zoonotic importance.

Currently *Babesia vogeli*, *B. canis* and *Babesia rossi* are categorized as the large species of canine *Babesia* (Panti‐May & Rodiguez‐Vivas, 2020). These three species that previously were described as sub‐species are distinct genetically and also differ in the severity of clinical symptoms that they cause, their tick vectors and geographic distribution (Depoix et al., 2002; Solano‐Gallego & Baneth, 2011; Zahler et al., 1998). Until now, three small species of *Babesia*, including *B. gibsoni*, *B. conradae* and *B. vulpes*, are recognized to infect dogs (Teodorowski et al., 2022). The occurrence of non‐canine *Babesia* (*B. caballi*), which is most dominantly found in horses is confirmed to infect dogs (Beck et al., 2009).

Although there have been recent studies estimating the regional and global prevalence of babesiosis, there is no comprehensive study for the disease in dogs on a worldwide scale. Therefore, this review and meta‐analysis aimed to estimate the global prevalence of babesiosis in dogs and assess the associated risk factors.

## 2 MATERIALS AND METHODS

### 2.1 Search strategy

The present study was performed according to the Preferred Reporting Items for Systematic Reviews and Meta‐Analysis checklist (PRISMA) (Page et al., 2021). The searching process was performed using multiple databases (Scopus, PubMed, ProQuest, Web of Science, and Google Scholar).Moreover, a hand search was carried out for the articles that were published from 2000 until December 2022. Search terms using AND and/or OR Boolean operators were as follows: *Babesia* spp., Babesiosis, *B. vogeli*, *B. canis*, *B. rossi*, *B. gibsoni*, *B. conradae*, *B. vulpes*, *B. caballi*, tick‐borne protozoan diseases, tick‐borne pathogens, blood protozoan parasites, dog, puppies, prevalence, frequency, global, worldwide. In addition to removing duplicates and irrelevant papers, the reference lists of the collected publications were checked for further studies that could not be located through database searches. The assessment of full‐text articles as well as the screening of the titles and abstracts of each article was performed independently by two authors.

### 2.2 Screening and eligibility of the study

All retrieved articles were primarily imported into the EndNote citation manager software (version 8, Thomson Reuters, Stamford, CT, USA) to sort and eliminate the duplicates. A Microsoft Excel version 2016 was applied to collect the following details from retrieved articles: first author's name, year of publication, countries, continent, time of sampling, sample size, number of positive samples, type of *Babesia*, climate (https://www.britannica.com/science/Koppen‐climate‐classification), seasons, diagnostic method (s) and stray/animal shelter dogs (Table S1, Figure S4 and Table 1).

**TABLE 1:** Sub‐group analysis based on seasons, diagnostic method, and stray/animal shelter dogs in included studies.

Variables	No studies	Sample size	Infected	Pooled prevalence (95% CI)	Heterogeneity		
					*I^2^*	*τ* ^2^	*p*‐Value
**Seasons**							
Spring	12	13704	551	0.080 (0.030–0.149)	95	0.034	<0.001
Summer	10	12367	396	0.097 (0.040–0.174)	97	0.030	<0.001
Autumn	9	12511	363	0.085 (0.022–0.179)	95	0.042	<0.001
Winter	10	12252	527	0.089 (0.023–0.188)	96	0.049	<0.001
**Diagnostic method**							
Blood smear	36	54871	3322	0.128 (0.074–0.194)	98	0.073	<0.001
ELISA	8	4086	406	0.108 (0.069–0.154)	89	0.008	<0.001
IFA	18	11617	1245	0.198 (0.115–0.297)	99	0.059	<0.001
PCR	110	44502	4054	0.117 (0.088–0.149)	98	0.059	<0.001
**Stray/animal shelter dogs**							
Stray/animal shelter dogs	27	5302	459	0.116 (0.066–0.178)	95	0.044	<0.001
Owned dogs	76	28113	3306	0.139 (0.104–0.180)	98	0.054	<0.001

Abbreviations: ELISA, enzyme‐linked immunosorbent assays; IFA, indirect fluorescent antibody.

### 2.3 Inclusion and exclusion criteria

The inclusion criteria considered for the current study were as follows: (1) All published observational studies (cross‐sectional, case–control and cohort) reporting the prevalence of *Babesia* in dogs, (2) availability of full‐text and abstract in English, (3) peer‐reviewed original articles, (4) availability of data regarding the total sample size and the number of positive cases, (5) articles published until December 2022. Those studies with sample size lower than 30, papers with non‐original data, review articles, case reports, case series, letters, editorials, publications with unclear or undetermined results were excluded from the analysis of the present study. Furthermore, those papers that reported *Babesia* infection in humans and in animals other than dogs were excluded.

### 2.4 Quality assessment

The included studies were evaluated for quality using the Newcastle‐Ottawa Scale (Table S2) (Eslahi et al., 2023; Modesti et al., 2016). Three factors made up the scoring system: selection (maximum of five stars), comparability (maximum of two stars) and result (maximum of three stars).

### 2.5 Data synthesis and statistical analysis

The global pooled prevalence of *Babesia* in dogs was estimated with a 95% confidence interval. To estimate the pooled prevalence, a Freeman–Tukey double arcsine transformation was applied using a random‐effects model. We employed Begg's rank test to identify potential publication bias. Additionally, publication bias was assessed using the Luis Furuya‐Kanamori (LFK) index and the Doi plot (Barendregt & Doi, 2016). In order to specify the impact of year of publication on the prevalence, a meta‐regression analysis was applied. An LFK index within the range of outside ±2, ±2 and ±1 is regarded as significantly/major asymmetrical, slightly/minor asymmetrical and asymmetrical symmetrical (absence of publication bias), respectively. A Freeman–Tukey double arcsine transformation for the random‐effects model was applied to calculate the overall prevalence. In order to assess the magnitude of heterogeneity among included studies, cochrane's *Q* test and inconsistency index (*I* ^2^ statistics) was used considering *I* ^2^ values of 25, 50 and 75% as low, medium and high heterogeneity, respectively. A *p*‐value lower than 0.05 was interpreted as statistically significant. All statistical analyses conducted herein were based on meta‐package of R (version 3.6.1) (Team, 2020).

## 3 RESULTS

### 3.1 Literature search selection and data extraction

**FIGURE 1:**
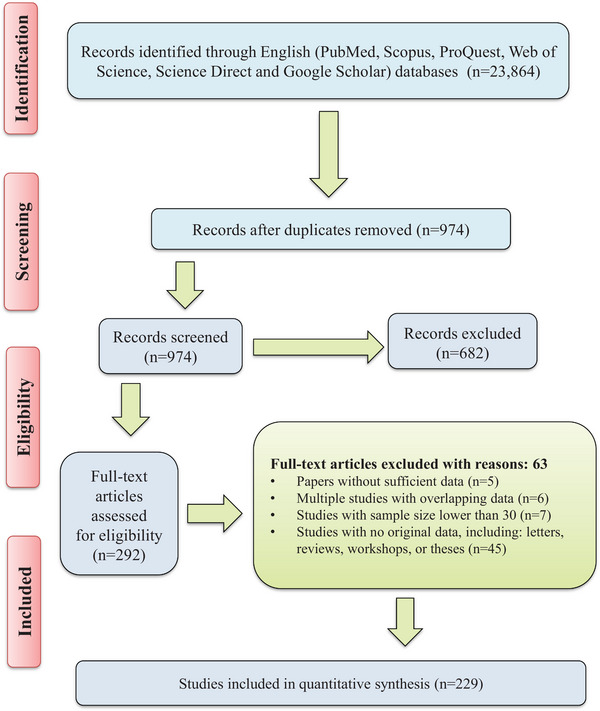
Flow diagram of the study design process.

The systematic search performed in the current study yielded a total of 23,864 articles. Totally, 292 full‐text papers were considered to be evaluated for eligibility. Among these publications, we excluded 5 studies due to insufficient data, 6 studies with overlapping data, 7 studies with sample size lower than 30 and 45 studies with no original data, including letters, reviews, workshops and theses. Finally, we included 229 papers, which were eligible according to the critical appraisal criteria (Figure 1).

### 3.2 Pooled prevalence

**FIGURE 2:**
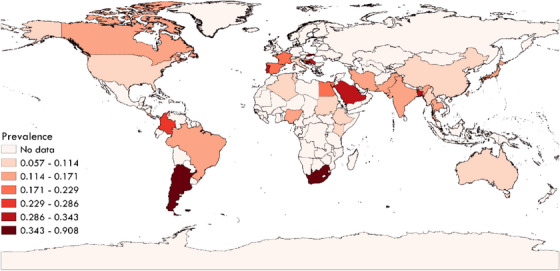
Global prevalence of *Babesia* in dogs in different geographical regions of the world based on the included studies.

The global pooled prevalence of *Babesia* in dogs was 0.120 (95% CI; 0.097–0.146) with a higher estimated pooled prevalence in owned dogs (0.139, 95% CI; 0.104–0.180) than stray/shelter dogs (0.116, 95% CI; 0.066–0.178) (Figure S1 and Table 1). The prevalence of *Babesia* in dogs has been documented in 61 countries of the world. A highest number of publications were related to India (29 studies). Our country‐based analysis showed that Slovakia (0.908, 95% CI; 0.826–0.948) following Bosnia and Herzegovina (0.825, 95% CI; 0.730–0.897) showed the highest pooled prevalence (Figure S2). A map was created using QGIS3 software (https://qgis.org/en/site/) to demonstrate the prevalence of *Babesia* in dogs in different geographical regions of the world (Figure 2).

The continent‐based estimates ranged from 0.207% to 0.074% that included a prevalence rate of 0.207 (99% CI; 0.097–0.344) for Europe, 0.135 (95% CI; 0.079–0.202) for South America, 0.104 (95% CI; 0.065–0.152) for Africa, 0.103 (95% CI; 0.062–0.154) for North America, 0.097 (95% CI; 0.072–0.126) for Asia and 0.074 (95% CI; 0.043–0.112) for Oceania (Figure S3). The present study showed that the highest pooled prevalence was attributable to the regions with tropical wet climate (0.156, 95% CI; 0.081–0.249), especially in summer (0.097, 95% CI; 0.040–0.174) (Figure S4 and Table 1). The global pooled prevalence of *Babesia* in dogs based on species or genus was as follows; 0.216 (95% CI; 0.056–0.441) for *B. canis*, 0.101 (95% CI; 0–0.677) for *B. conradea*, 0.089 (95% CI; 0.021–0.193) for *B. rossi*, 0.088 (95% CI; 0.065–0.113) for *B. gibsoni*, 0.063 (95% CI; 0.045–0.083) for *B. vogeli*, 0.035 (95% CI; 0.001–0.105) for *B. vulpes* and 0.005 (95% CI; 0–0.019) for *B. caballi* (Figure S5).

The estimated pooled prevalence based on reports of *Babesia* spp. in dogs was 0.123 (95% CI; 0.093–0.155) (Figure S6). The prevalence of *Babesia* spp. in dogs in different continents ranged from 0.189 to 0.023 including 0.189 (95% CI; 0.101–0.296) for South America, 0.154 (95% CI; 0.094–0.225) for Africa, 0.149 (95% CI; 0.090–0.219) for Europe, 0.133 (95% CI; 0.063–0.223) for Oceania, 0.058 (95% CI; 0.026–0.101) for Asia and 0.023 (95% CI; 0.001–0.063) for North America (Figure S7).

The highest pooled prevalence based on the detection method was related to the studies that utilized indirect fluorescent antibody (IFA) (0.198, 95% CI; 0.115–0.297) (Table 1).

### 3.3 Meta‐regression, publication bias and quality assessment

The meta‐regression analysis indicated that there was a statistically significant correlation between prevalence and year of publication (slop: 15.93, *p* < 0.05) (Figure S8).

There was a major asymmetry in the Doi plot (LFK index: 3.04). Furthermore, a highly significant publication bias was detected using the Linear regression plot (*t* = 6.03, *p* < 0.0001) (Figure S9).

The finding of the quality assessment indicated that, among 229 studies, 46 had a total score of 4–6 points (moderate level), and 183 had a total score of 7–9 points (high level) (Table S2).

## 4 DISCUSSION

The current systematic review and meta‐analysis study brings together, for the first time, true global prevalence data on *Babesia* parasites in dogs.

Slovakia followed by Bosnia and Herzegovina were the regions with highest prevalence of babesiosis in dogs, which was expected, as previous investigations indicated that *B. canis* is the commonly detected species in symptomatic dogs in European regions (Ćoralić et al., 2018). However, these estimates based on the country presented herein must be interpreted cautiously as they were related to single study for each country.

Babesiosis is a serious infection in dogs in subtropical and tropical regions (Kuo et al., 2020). *B. canis* occurs in the countries with a temperate climate, especially as Europe (Aktas et al., 2015; Øines et al., 2010). Similarly, the findings of the meta‐analysis presented herein suggested that climatic conditions have a substantial role in the prevalence of babesiosis in dogs with the highest pooled prevalence in regions with a tropical wet climate. Temperature and humidity are the potential factors that are advantageous to cause increased prevalence in tropical regions, as higher humidity and temperature are favorable to the development of life cycle of both vectors and parasites.

*B. canis*, the most prevalent parasite observed in the study, is the cause of a great number of clinical babesiosis cases in dogs in Europe (Solano‐Gallego et al., 2016). The clinical presentations associated with this species may vary from mild transient disease to acute illness with potential of mortality. Most of the canine babesiosis cases occurred in the central Europe revealed that they were complicated with a high mortality rate (Matijatko et al., 2012).

Europe was the continent with highest pooled prevalence. *B. canis* is endemic in the central Europe, and Baltic region. Germany and Poland from central Europe as well as Lithuania and Latvia from Baltic region are the newly documented endemic regions for canine babesiosis (Berzina et al., 2013; Paulauskas et al., 2014; Schäfer et al., 2021; Seleznova et al., 2020).

However, in central and northeast European regions, the disease is an emerging one (Bajer, Beck, et al., 2022; Pawełczyk et al., 2022). The geographical distribution of *Babesia* parasites in these regions is remarkably diverse, which highly depends on the distribution of the vector, the type of detection techniques, the species of *Babesia*, the country and cases under investigation (Solano‐Gallego et al., 2016). The fast growth of tourism with companion animals, particularly domestic dogs and cats, might have also contributed to the recent trend of increasing the prevalence of canine babesiosis in Europe. Moreover, it can be due to the diagnostic methods with higher sensitivity (e.g. molecular‐based techniques), which are applied in veterinary clinics (Pawełczyk et al., 2022). In European regions, the *Dermacentor reticulatus* is the vector for transmission of *Babesia* species in dogs and the spread of *B. canis* to new regions have a significant relationship with the wide distribution of this tick species (Drehmann et al., 2020; Dwużnik‐Szarek et al., 2021; Hornok et al., 2016; Rubel et al., 2016).

Higher prevalence of the infection was observed in dogs in summer. The peak activity of *D. reticulatus* is at the end of the spring and autumn. However, a highest number of clinical cases of the infection were reported in spring in Central Europe (Hornok et al., 2016). It is proposed that in environments with similar ecological features, the seasonal pattern of canine babesiosis is not solely determined by the availability of the appropriate vector. It is also affected by the early activity of ticks infected with *Babesia* (Hornok et al., 2016). In addition, *D. reticulatus* was also known to exist in western Slovakia (Majlathova et al., 2011), the country with the highest pooled prevalence for canine babesiosis in our study. The vectors responsible for the transmission of the *Babesia* species are as follows; *Haemaphysalis elliptica* (and probably *H. leachi*) for *B. rossi* (Penzhorn, 2020; Penzhorn et al., 2020), *Rhipicephalus sanguineus* for *B. vogeli* (Penzhorn, 2020), *H. longicornis* for *B. gibsoni* (Liu et al., 2018) and probably *D. reticulatus* for *B. caballi* (Daněk et al., 2022). Further studies are required for the identification of potential vector of *B. conradae*. However, the parasite was detected in the salivary glands of *R. sanguineus* (Dear et al., 2018; Liu et al., 2018).

The recent changes in the spread of *D. reticulatus* to new endemic regions of the world apparently reflects the important role of climate change and the possibility that specific local climatic conditions are responsible for the abrupt seasonal variations that changes the incidence of babesiosis in dogs (Leschnik et al., 2008). South America that showed the highest prevalence rate showcases a range of weather and climate conditions, incorporating tropical, subtropical and extratropical characteristics (Garreaud et al., 2009).

The occurrence of *Babesia* spp. in dogs of Latin America and the Caribbean exhibited considerable variability. The prevalence rates were 1.4% in Peru (Temoche et al., 2018), 2.2% in Venezuela (Criado‐Fornelio et al., 2007), 3.3% in Brazil (Silva et al., 2012), 5.5% in Colombia (Vargas‐Hernández et al., 2012) and 7.7% in Argentina (Mascarelli et al., 2016). In contrast, higher prevalence rates were reported in Nicaragua (15.4%) (Wei et al., 2014) and Brazil (23.4%) (Jojima, 2008). The lack of awareness regarding animal welfare and disease issues, economic constraints leading to restricted access to proper veterinary care and the absence of responsible practices in pet ownership are the factors that facilitated the transmission and persistence of tick‐borne diseases in this region. Moreover, socioeconomic and ecological elements, such as globalization, the rise in international trade, tourism and travel, climate change impacts, heightened mobility of dogs, alterations in landscape use and interactions with wildlife, have altered both the distribution of ticks and the patterns of infection for canine babesiosis (Panti‐May & Rodiguez‐Vivas, 2020).

The overall prevalence of *Babesia* parasites was higher in owned dogs than in stray/shelter dogs. This could be attributed to the comparatively higher number of studies documenting infections in owned dogs. Furthermore, the unrestrained access of owned dogs to public areas, infrequent application of ectoparasiticides and the advanced monitoring of owned dogs are among the factors that could potentially raise the detection rate and reduce the underestimation of infection cases in these animals compared to stray dogs.

Babesiosis is generally associated with fever, mild‐to‐severe anemia and thrombocytopenia, enlarged lymph nodes and spleen, jaundice and pigmenturia. The range of clinical signs and the severity of the disease is highly related to the species of *Babesia* causing infection along with the other factors such as age, concurrent infection and immune‐compromised situation (e.g. splenectomy and immunosuppressive treatment). The regular manifestations in canine babesiosis are anorexia, lethargy, weakness, pale mucous membranes, hypoalbuminemia and hyperbilirubinemia (Irwin, 2009; Solano‐Gallego et al., 2016; Solano‐Gallego & Baneth, 2011).

In our findings, the highest prevalence was related to IFA method. Despite the probable occurrence of cross‐reactivity between different *Babesia* species and other protozoa, IFA and the enzyme‐linked immunosorbent assays (ELISA) are commercially available and frequently utilized for diagnosis of babesiosis in dogs (Solano‐Gallego & Baneth, 2011). The assessment through IFA identifies antibodies against *Babesia* in the blood samples of animals that either are infected or have been exposed to the pathogen. It stands out as the most sensitive indirect approach for identifying occult and chronic babesiosis, as well as instances of low‐level parasitemia (Bicalho et al., 2004; Hartmann et al., 2013). Despite the appropriate sensitivity and ease of application provided by IFA, its specificity is reduced (Alvarez et al., 2019). The absence of standardized antigenic targets, potential cross‐reactivity and the difficulties in determination of positivity thresholds are the drawbacks (Garcia et al., 2022). For the accurate detection of *B. canis*, rBcMSA1 and rBcSA1 exhibit promising serodiagnostic antigens for indirect ELISA and rapid immunochromatographic tests (ICTs) (Zhou et al., 2016). Moreover, thrombospondin‐related adhesive protein (TRAP) is a recombinant protein derived from *B. gibsoni* can be used as an alternative for whole parasite antigen due to its high sensitivity and specificity (Solano‐Gallego & Baneth, 2011). Among the different molecular approaches, cPCR, nested PCR and multiplex PCR assays are considered as main methods with regard to the quality in detection of DNA in blood samples along with a newly customized portable real‐time PCR platform (Galon et al., 2022; Kuo et al., 2020).

## 5 LIMITATIONS

The present study has the following limitations:

1. The analyses presented herein may have been impacted by publication bias due to the lack of data or the limited number of published literatures from some geographic regions.
2. The present study was limited to publications in English language.
3. A significant number of studies included herein, used direct blood smear as a diagnostic method for detecting parasites, which may be associated with lower sensitivity and specificity and a high number of reports that did not specifically detect.
4. The parasite at the species level.

Despite these limitations, the current study provides the most comprehensive estimates of the prevalence of *Babesia* in dogs from a global perspective.

## 6 CONCLUSION

Understanding and addressing the risk factors associated with *Babesia* in dogs are crucial for an effective prevention and management of the infection. Geographical location can increase the risk of infection in dogs, as certain regions have a higher prevalence of ticks carrying *Babesia* parasites.

The findings of the present study underscore the need for investigations in a broader range of geographical areas. Dogs in regions with a warm humid continental climate showed a higher prevalence of the infection, emphasizing the necessity of sufficient strategies for animal health and biosecurity measures in these regions. The infection was most prevalent in owned dogs, which is crucial for owners to implement effective tick prevention measures, maintain awareness of regional risks, and seek veterinary care promptly if any symptoms of babesiosis are observed. Regular check‐ups and consultations with veterinarians can also contribute to the early detection and management of the disease. The limitations of diagnostic techniques and the standardization of current methods must also be taken into account by reference laboratories. The surveillance and control sectors should consider the priorities of each region, as they may have substantial differences between developed and developing countries.

## AUTHOR CONTRIBUTIONS

*Conceptualization*: Aida Vafae Eslahi, Milad Badri and Panagiotis Karanis. *Data curation*: Meysam Olfatifar, Aida Vafae Eslahi and Milad Badri. *Formal analysis*: Meysam Olfatifar. *Funding acquisition*: Milad Badri. *Investigation*: Amir Abdoli, Leila Zaki, Behzad Bijani, Majid Pirestani and Kareem Hatam‐Nahavandi. *Methodology*: Meysam Olfatifar, Aida Vafae Eslahi, Leila Zaki and Milad Badri. *Project administration*: Amir Abdoli, Aida Vafae Eslahi, Milad Badri and Panagiotis Karanis. *Software*: Meysam Olfatifar. *Supervision*: Amir Abdoli, Milad Badri and Panagiotis Karanis. *Validation*: Meysam Olfatifar and Milad Badri. *Visualization*: Milad Badri and Panagiotis Karanis. *Writing—original draft*: Amir Abdoli, Aida Vafae Eslahi, Milad Badri and Panagiotis Karanis. *Writing—review and editing*: Aida Vafae Eslahi, Milad Badri and Panagiotis Karanis.

## CONFLICT OF INTEREST STATEMENT

The authors declared no potential conflicts of interest concerning the research or authorship.

## FUNDING INFORMATION

Medical Microbiology Research Center, Qazvin University of Medical Sciences, Qazvin, Iran, under the contract no.: IR.QUMS.REC.1401.351

## ETHICS STATEMENT

Ethical approval was required and provided for this study, as stated by our institutional review board.

### PEER REVIEW

The peer review history for this article is available at https://publons.com/publon/10.1002/vms3.1427.

## Supporting information

Supporting information

Supporting information

Supporting information

Supporting information

Supporting information

Supporting information

Supporting information

Supporting information

Supporting information

Supporting information

Supporting information

## Data Availability

All data are included in the manuscript or as supplementary files.
