# Supplementary material for: A global systematic review and meta‐analysis on the babesiosis in dogs with special reference to Babesia canis
Source: Vet Med Sci. 2024 May 2;10(3):e1427. doi: 10.1002/vms3.1427 (PMC11063922; doi:10.1002/vms3.1427)
Supplement: Supplementary file 1 — Supporting information [file VMS3-10-e1427-s005.pdf]

Studies	Prevalence	95% CI
Macintire, et al. 2002 U.S.A	0.1500	[0.0970; 0.2247]
Birkenheuer, et al. 2003 U.S.A	0.0846	[0.0634; 0.1121]
Inokuma, et al. 2004 Japan	0.1500	[0.0879; 0.2441]
Song, et al. 2004 South Korea	0.0180	[0.0095; 0.0338]
Matsuu, et al. 2004 Japan	0.2979	[0.2286; 0.3779]
Ikadai, et al. 2004 Japan	0.0481	[0.0368; 0.0625]
Verdida, et al 2004 Japan	0.1689	[0.1251; 0.2242]
Verdida, et al 2004 China	0.0488	[0.0135; 0.1614]
Oyamada, et al. 2005 Sudan	0.0897	[0.0442; 0.1738]
Birkenheuer, et al. 2005 U.S.A	0.2140	[0.1847; 0.2465]
Miyama, et al. 2005 Japan	0.2435	[0.1925; 0.3029]
Gary, et al. 2006 Canada	0.1250	[0.0917; 0.1682]
Trapp, et al. 2006 Brazil	0.3570	[0.3105; 0.4063]
Criado, et al. 2006 Spain	0.2100	[0.1417; 0.2998]
Jefferies, et al. 2007 Australia	0.0927	[0.0649; 0.1307]
Maia, et al. 2007 Brazil	0.1881	[0.1564; 0.2245]
Cruz-Flores, et al. 2008 Brazil	0.2708	[0.1657; 0.4100]
Konishi, et al. 2008 Japan	0.1061	[0.0900; 0.1248]
M'ghirbi and Bouattour. 2008 Tunisia	0.0667	[0.0385; 0.1129]
Matjila, et al. 2008 South Africa	0.3849	[0.3570; 0.4135]
Solano-Gallego, et al. 2008 Italy	0.2744	[0.2119; 0.3473]
Yabsley, et al. 2008 Grenada	0.0685	[0.0296; 0.1505]
Beck, et al. 2009 Croatia	0.1173	[0.0982; 0.1396]
Cassini, et al. 2009 Italy	0.1249	[0.1072; 0.1450]
Costa-Júnior, et al. 2009 Brazil	0.2869	[0.2338; 0.3466]
Criado-Fornelio, et al. 2009 France	0.0093	[0.0016; 0.0506]
Tabar, et al. 2009 Spain	0.0588	[0.0313; 0.1080]
Yeagley, et al. 2009 U.S.A	0.3376	[0.2683; 0.4146]
Wu, et al. 2009 Taiwan	0.0693	[0.0340; 0.1362]
Lee, et al. 2010 Taiwan	0.2320	[0.1667; 0.3133]
Fritz. 2010 France	0.6506	[0.5754; 0.7190]
Ramos, et al. 2010 Brazil	0.0341	[0.0166; 0.0688]
Wang, et al. 2010 U.S.A	0.1064	[0.0731; 0.1523]
Cardoso, et al. 2010 Portugal	0.8000	[0.6618; 0.8910]
Levy, et al. 2011 U.S.A	0.0205	[0.0099; 0.0416]
Abd Rani, et al. 2011 India	0.0190	[0.0104; 0.0347]
Varanat, et al. 2011 U.S.A	0.0073	[0.0013; 0.0402]
Majlathova, et al. 2011 Slovakia	0.9080	[0.8289; 0.9527]
Buddhachat, et al. 2012 Thailand	0.2255	[0.1552; 0.3157]
Costa-Júnior, et al. 2012 Brazil	0.1029	[0.0773; 0.1357]
Singh, et al. 2012 India	0.0891	[0.0664; 0.1187]
Hii, et al. 2012 Australia	0.0565	[0.0333; 0.0943]
Vargas-Hernández, et al. 2012 Colombia	0.2857	[0.2250; 0.3552]
Kamani, et al. 2013 Nigeria	0.0331	[0.0153; 0.0704]
Kelly, et al. 2013 Saint Kitts and Nevis	0.1290	[0.0987; 0.1669]
Loftis, et al. 2013 Saint Kitts and Nevis	0.1515	[0.1048; 0.2141]
Adamu, et al. 2014 Nigeria	0.3100	[0.2278; 0.4063]
Laha, et al. 2014 India	0.1892	[0.1431; 0.2458]
Rojas, et al. 2014 Costa Rica	0.0822	[0.0476; 0.1382]
Singh, et al. 2014 India	0.1145	[0.0877; 0.1481]
Giudice, et al. 2015 Italy	0.0116	[0.0021; 0.0630]
Terao, et al. 2015 Bangladesh	0.3000	[0.1910; 0.4375]
Minervino, et al. 2015 Brazil	0.1407	[0.1071; 0.1825]
Cao, et al. 2015 China	0.0923	[0.0770; 0.1103]
Xu, et al. 2015 China	0.0117	[0.0068; 0.0199]
Araujo, et al. 2015 Brazil	0.5792	[0.5305; 0.6264]
Davitkov, et al. 2015 Serbia	0.9833	[0.9413; 0.9954]
El-Dakhly, et al. 2015 Japan	0.2219	[0.1836; 0.2657]
Gabrielli, et al. 2015 Serbia	0.1139	[0.0733; 0.1729]
Krawczak, et al. 2015 Brazil	0.3125	[0.2285; 0.4109]
Kubo, et al. 2015 Japan	0.0222	[0.0137; 0.0357]
Miró, et al. 2015 Spain	0.4667	[0.4046; 0.5298]
Piratae, et al. 2015 Thailand	0.0633	[0.0273; 0.1397]
René-Martellet, et al. 2015 France	0.1357	[0.0886; 0.2023]
Vipan, et al. 2015 India	0.0784	[0.0489; 0.1236]
Singh, et al. 2016 India	0.0793	[0.0469; 0.1309]
Bhaskaran Ravi, et al. 2016 India	0.0412	[0.0390; 0.0436]
Akhtardanesh, et al. 2016 Iran	0.0500	[0.0171; 0.1370]
da Silva, et al. 2016 Brazil	0.0479	[0.0234; 0.0957]
Hamel, et al. 2016 Albania	0.0299	[0.0190; 0.0468]
Mascarelli, et al. 2016 Argentina	0.0429	[0.0147; 0.1186]
Rjeibi, et al. 2016 Tunisia	0.1250	[0.0861; 0.1780]
Singla, et al. 2016 India	0.1530	[0.1294; 0.1799]
Starkey, et al. 2016 Haiti	0.0386	[0.0197; 0.0744]
Zheng, et al. 2017 China	0.0741	[0.0429; 0.1250]
Adao, et al. 2017 Philippin	0.0526	[0.0243; 0.1101]
Aktas and Ozubek. 2017 Turkey	0.0183	[0.0071; 0.0460]
Andersson, et al. 2017 Romania	0.0104	[0.0018; 0.0567]
Augustine, al. 2017 India	0.4313	[0.3570; 0.5087]
Azmi, al. 2017 Palestine	0.0193	[0.0094; 0.0394]
Kebede and Dereje. 2017 Ethiopia	0.0599	[0.0402; 0.0883]
Ribeiro, et al. 2017 Brazil	0.1099	[0.0723; 0.1636]
Zhang, et al. 2017 China	0.0391	[0.0260; 0.0586]
Jikuya, et al 2017 Japan	0.0227	[0.0063; 0.0791]
Niu, et al 2017 China	0.0142	[0.0039; 0.0502]
Pavlović, et al. 2017 Serbia	0.1205	[0.0857; 0.1668]
Annoscia, et al. 2017 Italy	0.1020	[0.0628; 0.1615]
He, et al. 2017 China	0.1017	[0.0693; 0.1469]
Ahmad, et al. 2018 Pakistan	0.3356	[0.3055; 0.3670]
Filipović, et al. 2018 Serbia	0.0270	[0.0092; 0.0765]
Kirade, et al. 2018 India	0.0333	[0.0143; 0.0757]
Kushwaha, et al. 2018 India	0.0165	[0.0146; 0.0188]
Jain, et al. 2018 India	0.3167	[0.2666; 0.3713]
Temoche, et al. 2018 Peru	0.0142	[0.0048; 0.0408]
Ćoralić, et al. 2018 Bosnia and Herzegovina	0.8250	[0.7274; 0.8928]
Barash, et al. 2019 U.S.A	0.0217	[0.0189; 0.0248]
Wang, et al. 2019 China	0.0538	[0.0263; 0.1070]
Betgiri, et al. 2019 India	0.4400	[0.3630; 0.5200]
Inácio, et al. 2019 Paraguay	0.0547	[0.0360; 0.0822]
Divya, et al. 2019 India	0.1390	[0.1256; 0.1536]
Akram, et al. 2019 Pakistan	0.0133	[0.0037; 0.0473]
Bilgic, et al. 2019 Turkey	0.0211	[0.0107; 0.0411]
Braga, et al. 2019 Brazil	0.1852	[0.1617; 0.2112]
Checa, et al. 2019 Spain	0.1706	[0.1455; 0.1991]
Modarelli, et al. 2019 U.S.A	0.0043	[0.0018; 0.0100]
Teodorowski, et al. 2020 Poland	0.0139	[0.0047; 0.0400]
Hassanen. 2020 Egypt	0.1533	[0.1044; 0.2196]
Bahiense, et al. 2020 Brazil	0.0343	[0.0158; 0.0728]
Alanazi, et al. 2020 Saudi Arabia	0.3000	[0.2054; 0.4154]
Ngoka, et al. 2020 Kenya	0.0909	[0.0539; 0.1493]
Paschoal, et al. 2020 Brazil	0.1063	[0.0813; 0.1378]
Medkour, et al. 2020 Côte d'Ivoire	0.0081	[0.0014; 0.0446]
Badawi and Yousif. 2020 Iraq	0.0339	[0.0223; 0.0512]
Castro, et al. 2020 Brazil	0.0246	[0.0134; 0.0446]
Guo, et al. 2020 China	0.4717	[0.4215; 0.5225]
Kaur, et al. 2020 India	0.0062	[0.0024; 0.0159]
Kiouani, et al. 2020 Algeria	0.0582	[0.0387; 0.0865]
Lee, et al. 2020 Korea	0.0172	[0.0125; 0.0235]
Manoj, et al. 2020 India	0.1043	[0.0711; 0.1506]
Obeta, et al. 2020 Nigeria	0.1083	[0.0836; 0.1393]
Senthil, et al. 2020 India	0.0761	[0.0713; 0.0812]
Wang, et al. 2020 China	0.0087	[0.0015; 0.0476]
Zanet, et al. 2020 Italy	0.0399	[0.0310; 0.0512]
Li, et al. 2020 China	0.1103	[0.0784; 0.1531]
ASLANTAŞ, et al. 2020 Turkey	0.0108	[0.0030; 0.0384]
Díaz-Regañón, et al. 2020 Nepal	0.0286	[0.0079; 0.0983]
Habibi, et al. 2020 Iran	0.2500	[0.1419; 0.4019]
Zaki, et al. 2021 Egypt	0.2562	[0.2053; 0.3147]
Bouattour, et al. 2021 Tunisia	0.0303	[0.0104; 0.0853]
Kopparthi, et al. 2021 India	0.2002	[0.1752; 0.2279]
Ngoka, et al. 2021 Kenya	0.0909	[0.0539; 0.1493]
Nhuong, et al. 2021 Cambodia	0.0022	[0.0004; 0.0123]
García-Quesada, et al. 2021 Costa Rica	0.1747	[0.1507; 0.2016]
Panda, et al. 2021 India	0.1919	[0.1431; 0.2524]
Dordio, et al. 2021 Portugal	0.0211	[0.0072; 0.0603]
Stayton, et al. 2021 U.S.A	0.3750	[0.2422; 0.5297]
Carli, et al. 2021 Italy	0.0659	[0.0488; 0.0885]
Bawm, et al. 2021 Myanmar	0.0769	[0.0378; 0.1504]
Dos Santos, et al 2022 Brazil	0.0811	[0.0583; 0.1117]
Bordoloi, et al 2022 India	0.3317	[0.2709; 0.3987]
Fonsêca, et al 2022 Brazil	0.1242	[0.0810; 0.1858]
Niestat, et al 2022 U.S.A	0.1673	[0.1504; 0.1858]
Padmaja, et al 2022 India	0.0550	[0.0366; 0.0819]
Yang, et al 2022 Taiwan	0.1264	[0.1008; 0.1574]
Aitor, et al. 2022 Chile	0.0065	[0.0028; 0.0152]
**Random effects model**	**0.1207**	**[0.0970; 0.1465]**

Heterogeneity: $I^2 = 98.5638\%$, $\tau^2 = 0.0515$, $p < .001$

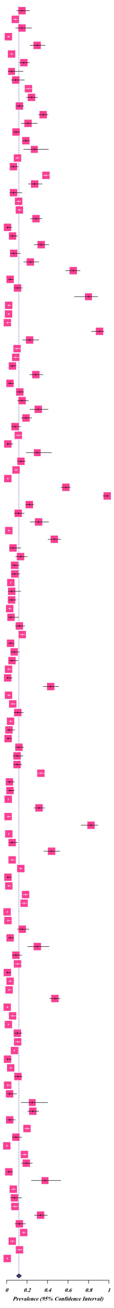

Supplementary Figure 1. Forest plots for random-effects meta-analysis of *Babesia* in dogs based on included studies (The boxes indicate the effect size of the studies (prevalence) and the whiskers indicate its confidence interval for corresponding effect size. There is no specific difference between white and black bars, only studies with a very narrow confidence interval are shown in white. In the case of diamonds, their size indicates the size of the effect, and their length indicate confidence intervals).
