# Supplementary figures and images for: A global systematic review and meta‐analysis on the babesiosis in dogs with special reference to Babesia canis
Source: Vet Med Sci. 2024 May 2;10(3):e1427. doi: 10.1002/vms3.1427 (PMC11063922; doi:10.1002/vms3.1427)

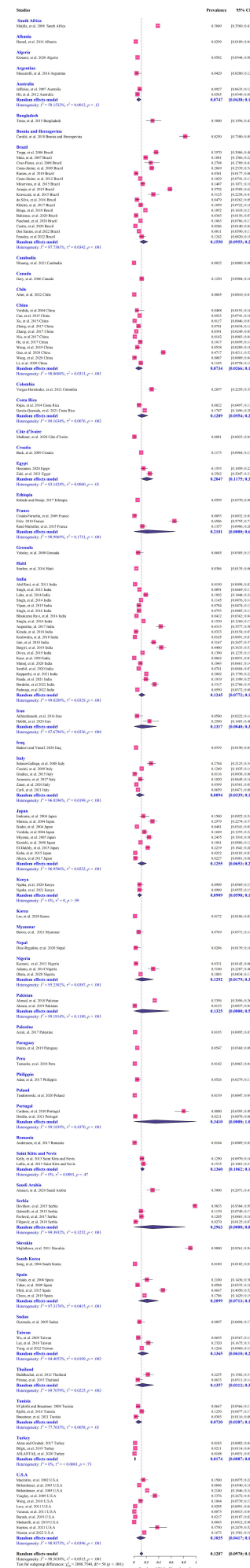

Supplement: Supplementary file 2 — Supporting information [file VMS3-10-e1427-s006.pdf]

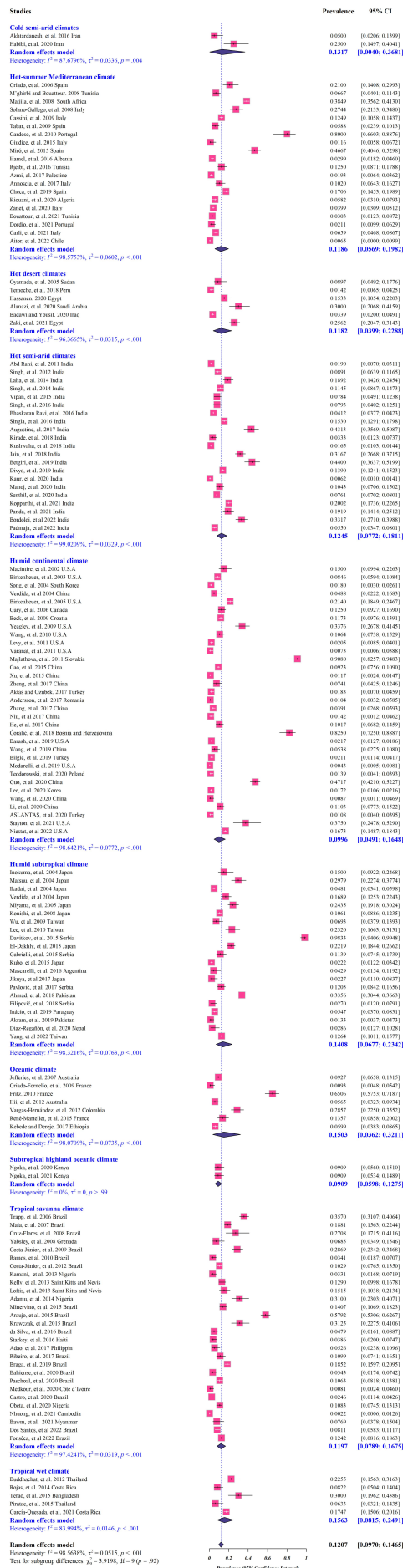

Supplement: Supplementary file 4 — Supporting information [file VMS3-10-e1427-s007.pdf]

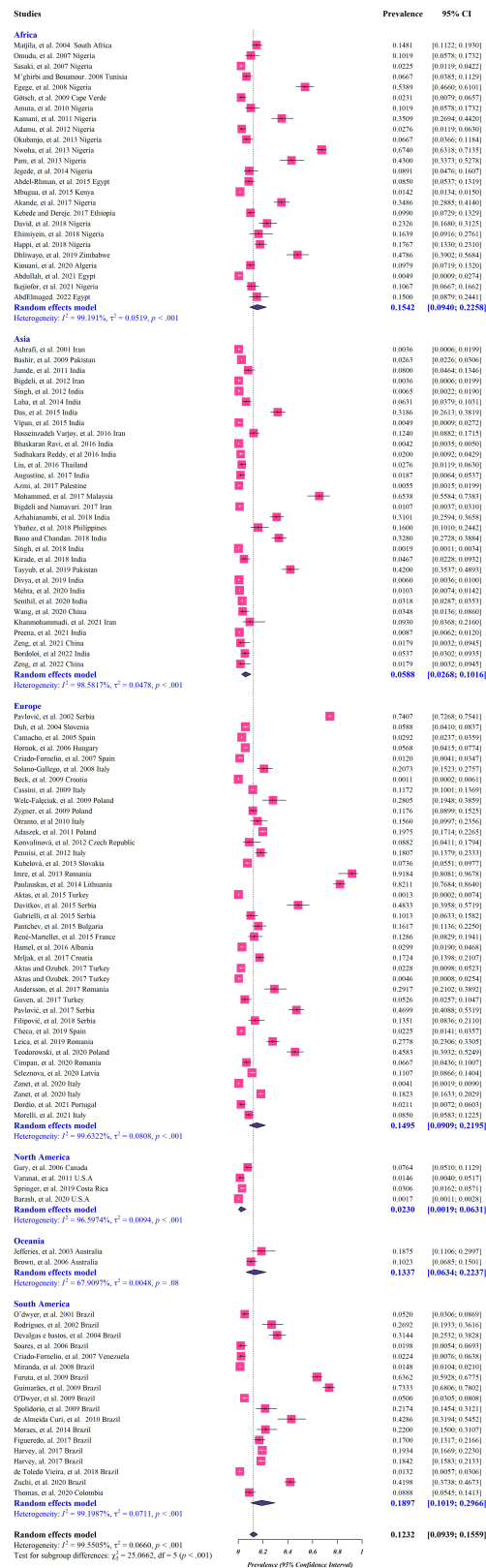

Supplement: Supplementary file 7 — Supporting information [file VMS3-10-e1427-s008.pdf]
