# Supplementary material for: A global systematic review and meta‐analysis on the babesiosis in dogs with special reference to Babesia canis
Source: Vet Med Sci. 2024 May 2;10(3):e1427. doi: 10.1002/vms3.1427 (PMC11063922; doi:10.1002/vms3.1427)
Supplement: Supplementary file 6 — Supporting information [file VMS3-10-e1427-s009.pdf]

Studies	Prevalence	95% CI
Ashrafi, et al. 2001 Iran	0.0036	[0.0006; 0.0199]
O'dwyer, et al. 2001 Brazil	0.0520	[0.0306; 0.0869]
Pavlović, et al. 2002 Serbia	0.7407	[0.7268; 0.7541]
Rodrigues, et al. 2002 Brazil	0.2692	[0.1933; 0.3616]
Jefferies, et al. 2003 Australia	0.1875	[0.1106; 0.2997]
Devalgas e bastos, et al. 2004 Brazil	0.3144	[0.2532; 0.3828]
Duh, et al. 2004 Slovenia	0.0588	[0.0410; 0.0837]
Matjila, et al. 2004 South Africa	0.1481	[0.1122; 0.1930]
Camacho, et al. 2005 Spain	0.0292	[0.0237; 0.0359]
Gary, et al. 2006 Canada	0.0764	[0.0510; 0.1129]
Brown, et al. 2006 Australia	0.1023	[0.0685; 0.1501]
Hornok, et al. 2006 Hungary	0.0568	[0.0415; 0.0774]
Soares, et al. 2006 Brazil	0.0198	[0.0054; 0.0693]
Criado-Fornelio, et al. 2007 Venezuela	0.0224	[0.0076; 0.0638]
Criado-Fornelio, et al. 2007 Spain	0.0120	[0.0041; 0.0347]
Omudu, et al. 2007 Nigeria	0.1019	[0.0578; 0.1732]
Sasaki, et al. 2007 Nigeria	0.0225	[0.0119; 0.0422]
M'ghirbi and Bouattour. 2008 Tunisia	0.0667	[0.0385; 0.1129]
Egege, et al. 2008 Nigeria	0.5389	[0.4660; 0.6101]
Solano-Gallego, et al. 2008 Italy	0.2073	[0.1523; 0.2757]
Miranda, et al. 2008 Brazil	0.0148	[0.0104; 0.0210]
Beck, et al. 2009 Croatia	0.0011	[0.0002; 0.0061]
Bashir, et al. 2009 Pakistan	0.0263	[0.0226; 0.0306]
Cassini, et al. 2009 Italy	0.1172	[0.1001; 0.1369]
Furuta, et al. 2009 Brazil	0.6362	[0.5928; 0.6775]
Götsch, et al. 2009 Cape Verde	0.0231	[0.0079; 0.0657]
Guimarães, et al. 2009 Brazil	0.7333	[0.6806; 0.7802]
O'Dwyer, et al. 2009 Brazil	0.0500	[0.0305; 0.0808]
Welc-Falęciak, et al. 2009 Poland	0.2805	[0.1948; 0.3859]
Zygner, et al. 2009 Poland	0.1176	[0.0899; 0.1525]
Spolidorio, et al. 2009 Brazil	0.2174	[0.1454; 0.3121]
de Almeida Curi, et al. 2010 Brazil	0.4286	[0.3194; 0.5452]
Otranto, et al 2010 Italy	0.1560	[0.0997; 0.2356]
Amuta, et al. 2010 Nigeria	0.1019	[0.0578; 0.1732]
Adaszek, et al. 2011 Poland	0.1975	[0.1714; 0.2265]
Jumde, et al. 2011 India	0.0800	[0.0464; 0.1346]
Kamani, et al. 2011 Nigeria	0.3509	[0.2694; 0.4420]
Varanat, et al. 2011 U.S.A	0.0146	[0.0040; 0.0517]
Bigdeli, et al. 2012 Iran	0.0036	[0.0006; 0.0199]
Singh, et al. 2012 India	0.0065	[0.0022; 0.0190]
Konvalinová, et al. 2012 Czech Republic	0.0882	[0.0411; 0.1794]
Pennisi, et al. 2012 Italy	0.1807	[0.1379; 0.2333]
Adamu, et al. 2012 Nigeria	0.0276	[0.0119; 0.0630]
Kubelová, et al. 2013 Slovakia	0.0736	[0.0551; 0.0977]
Imre, et al. 2013 Romania	0.9184	[0.8081; 0.9678]
Okubanjo, et al. 2013 Nigeria	0.0667	[0.0366; 0.1184]
Nwoha, et al. 2013 Nigeria	0.6740	[0.6318; 0.7135]
Pam, et al. 2013 Nigeria	0.4300	[0.3373; 0.5278]
Jegede, et al. 2014 Nigeria	0.0891	[0.0476; 0.1607]
Laha, et al. 2014 India	0.0631	[0.0379; 0.1031]
Moraes, et al. 2014 Brazil	0.2200	[0.1500; 0.3107]
Paulauskas, et al. 2014 Lithuania	0.8211	[0.7684; 0.8640]
Abdel-Rhman, et al. 2015 Egypt	0.0850	[0.0537; 0.1319]
Aktas, et al. 2015 Turkey	0.0013	[0.0002; 0.0074]
Das, et al. 2015 India	0.3186	[0.2613; 0.3819]
Davitkov, et al. 2015 Serbia	0.4833	[0.3958; 0.5719]
Gabrielli, et al. 2015 Serbia	0.1013	[0.0633; 0.1582]
Mbugua, et al. 2015 Kenya	0.0142	[0.0134; 0.0150]
Pantchev, et al. 2015 Bulgaria	0.1617	[0.1136; 0.2250]
René-Martellet, et al. 2015 France	0.1286	[0.0829; 0.1941]
Vipan, et al. 2015 India	0.0049	[0.0009; 0.0272]
Hosseinzadeh Varjoy, et al. 2016 Iran	0.1240	[0.0882; 0.1715]
Bhaskaran Ravi, et al. 2016 India	0.0042	[0.0035; 0.0050]
Sudhakara Reddy, et al 2016 India	0.0200	[0.0092; 0.0429]
Hamel, et al. 2016 Albania	0.0299	[0.0190; 0.0468]
Liu, et al. 2016 Thailand	0.0276	[0.0119; 0.0630]
Mrljak, et al. 2017 Croatia	0.1724	[0.1398; 0.2107]
Akande, et al. 2017 Nigeria	0.3486	[0.2885; 0.4140]
Aktas and Ozubek. 2017 Turkey	0.0228	[0.0098; 0.0523]
Aktas and Ozubek. 2017 Turkey	0.0046	[0.0008; 0.0254]
Andersson, et al. 2017 Romania	0.2917	[0.2102; 0.3892]
Augustine, al. 2017 India	0.0187	[0.0064; 0.0537]
Azmi, al. 2017 Palestine	0.0055	[0.0015; 0.0199]
Figueredo, al. 2017 Brazil	0.1700	[0.1317; 0.2166]
Guven, al. 2017 Turkey	0.0526	[0.0257; 0.1047]
Harvey, al. 2017 Brazil	0.1934	[0.1669; 0.2230]
Harvey, al. 2017 Brazil	0.1842	[0.1583; 0.2133]
Kebede and Dereje. 2017 Ethiopia	0.0990	[0.0729; 0.1329]
Mohammed, et al. 2017 Malaysia	0.6538	[0.5584; 0.7383]
Bigdeli and Namavari. 2017 Iran	0.0107	[0.0037; 0.0310]
Pavlović, et al. 2017 Serbia	0.4699	[0.4088; 0.5319]
Azhahianambi, et al. 2018 India	0.3101	[0.2594; 0.3658]
David, et al. 2018 Nigeria	0.2326	[0.1680; 0.3125]
Ybañez, et al. 2018 Philippines	0.1600	[0.1010; 0.2442]
Bano and Chandan. 2018 India	0.3280	[0.2728; 0.3884]
Ehimiyein, et al. 2018 Nigeria	0.1639	[0.0916; 0.2761]
Filipović, et al. 2018 Serbia	0.1351	[0.0836; 0.2110]
Happi, et al. 2018 Nigeria	0.1767	[0.1330; 0.2310]
Singh, et al. 2018 India	0.0019	[0.0011; 0.0034]
Kirade, et al. 2018 India	0.0467	[0.0228; 0.0932]
de Toledo Vieira, et al. 2018 Brazil	0.0132	[0.0057; 0.0306]
Tayyub, et al. 2019 Pakistan	0.4200	[0.3537; 0.4893]
Divya, et al. 2019 India	0.0060	[0.0036; 0.0100]
Checa, et al. 2019 Spain	0.0225	[0.0141; 0.0357]
Springer, et al. 2019 Costa Rica	0.0306	[0.0162; 0.0571]
Dhliwayo, et al. 2019 Zimbabwe	0.4786	[0.3902; 0.5684]
Leica, et al. 2019 Romania	0.2778	[0.2306; 0.3305]
Barash, et al. 2020 U.S.A	0.0017	[0.0011; 0.0028]
Zuchi, et al. 2020 Brazil	0.4198	[0.3738; 0.4673]
Teodorowski, et al. 2020 Poland	0.4583	[0.3932; 0.5249]
Mehta, et al. 2020 India	0.0103	[0.0074; 0.0142]
Cimpan, et al. 2020 Romania	0.0667	[0.0436; 0.1007]
Kiouani, et al. 2020 Algeria	0.0979	[0.0719; 0.1320]
Seleznova, et al. 2020 Latvia	0.1107	[0.0866; 0.1404]
Senthil, et al. 2020 India	0.0318	[0.0287; 0.0353]
Thomas, et al. 2020 Colombia	0.0888	[0.0545; 0.1413]
Wang, et al. 2020 China	0.0348	[0.0136; 0.0860]
Zanet, et al. 2020 Italy	0.0041	[0.0019; 0.0090]
Zanet, et al. 2020 Italy	0.1823	[0.1633; 0.2029]
Abdullah, et al. 2021 Egypt	0.0049	[0.0009; 0.0274]
Khanmohammadi, et al. 2021 Iran	0.0930	[0.0368; 0.2160]
Preena, et al. 2021 India	0.0087	[0.0062; 0.0120]
Zeng, et al. 2021 China	0.0179	[0.0032; 0.0945]
Dordio, et al. 2021 Portugal	0.0211	[0.0072; 0.0603]
Ikejiofor, et al. 2021 Nigeria	0.1067	[0.0667; 0.1662]
Morelli, et al. 2021 Italy	0.0850	[0.0583; 0.1225]
AbdElmaged. 2022 Egypt	0.1500	[0.0879; 0.2441]
Bordoloi, et al 2022 India	0.0537	[0.0302; 0.0935]
Zeng, et al. 2022 China	0.0179	[0.0032; 0.0945]
**Random effects model**	**0.1232**	**[0.0939; 0.1559]**

Heterogeneity: $I^2 = 99.5505\%$, $\tau^2 = 0.0660$, $p < .001$

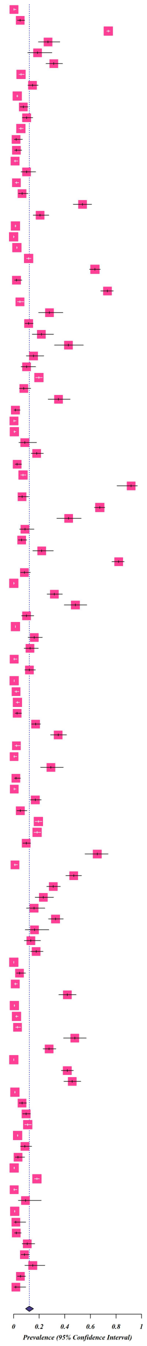

Supplementary Figure 6. Forest plots for random-effects meta-analysis of *Babesia* spp. in dogs based on the included studies (The boxes indicate the effect size of the studies (prevalence) and the whiskers indicate its confidence interval for corresponding effect size. There is no specific difference between white and black bars, only studies with a very narrow confidence interval are shown in white. In the case of diamonds, their size indicates the size of the effect, and their length indicate confidence intervals).
