# Supplementary material for: A global systematic review and meta‐analysis on the babesiosis in dogs with special reference to Babesia canis
Source: Vet Med Sci. 2024 May 2;10(3):e1427. doi: 10.1002/vms3.1427 (PMC11063922; doi:10.1002/vms3.1427)
Supplement: Supplementary file 8 — Supporting information [file VMS3-10-e1427-s010.pdf]

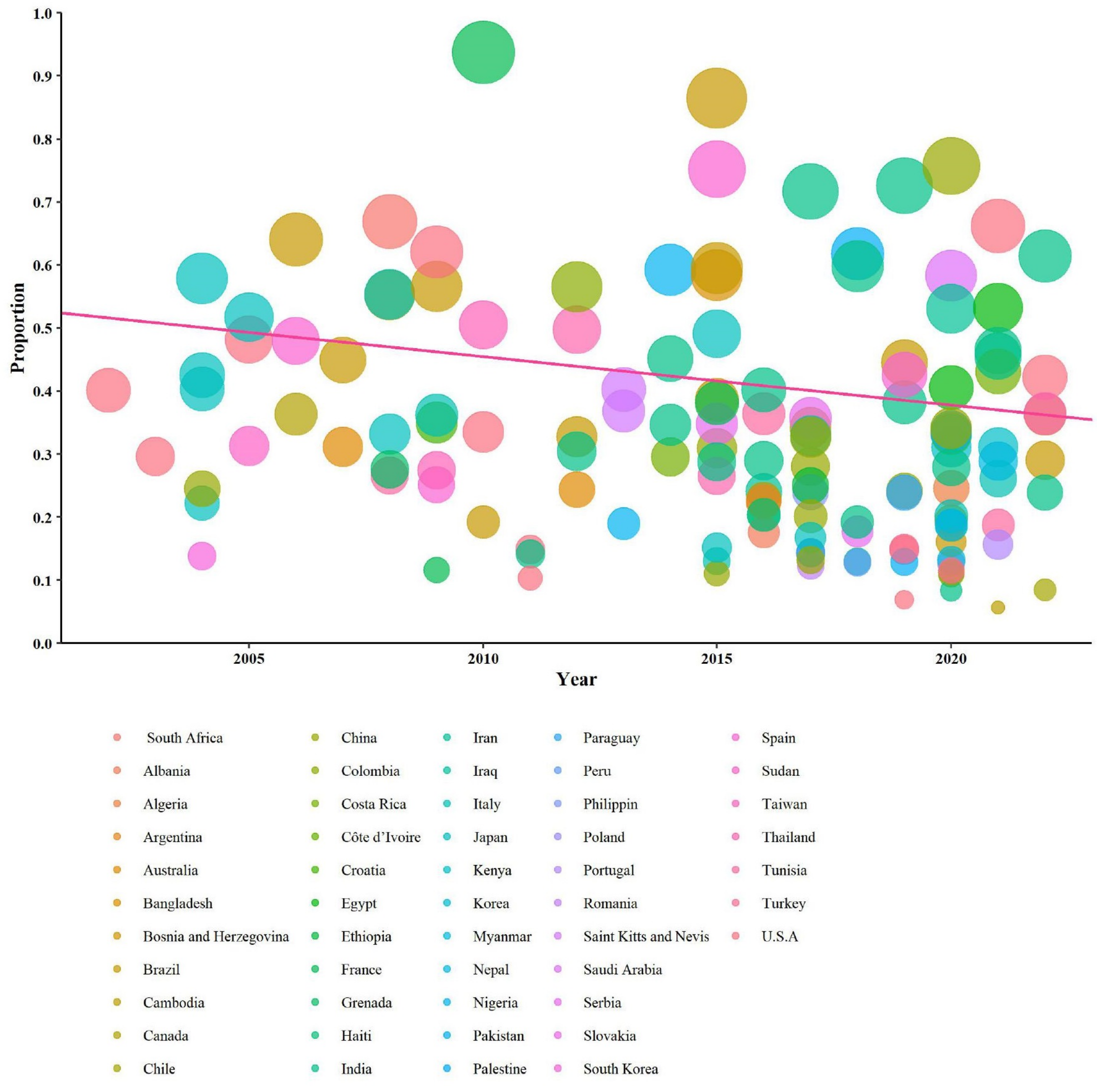

Supplementary Figure 8. Meta-regression graph for the prevalence of *Babesia* in dogs based on year of publication (The pink line is the regression line, which was plotted based on the intercept and the slope of the regression model. The different color bubbles represent the countries under study and their sizes indicate the effect size of each study).
