# Supplementary material for: A global systematic review and meta‐analysis on the babesiosis in dogs with special reference to Babesia canis
Source: Vet Med Sci. 2024 May 2;10(3):e1427. doi: 10.1002/vms3.1427 (PMC11063922; doi:10.1002/vms3.1427)
Supplement: Supplementary file 9 — Supporting information [file VMS3-10-e1427-s004.pdf]

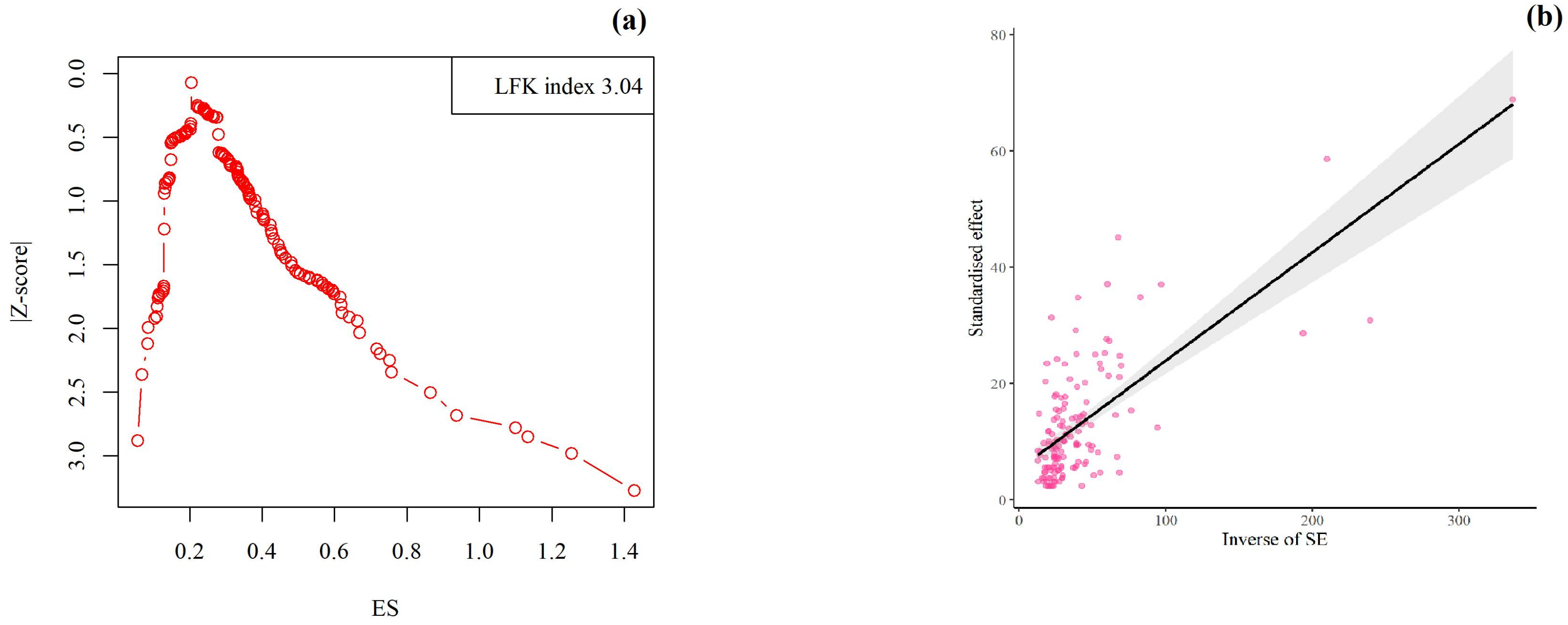

Supplementary Figure 9. (**a**) Doi plot of global prevalence of *Babesia* in dogs, A Luis Furuya -Kanamori (LFK) index 3.04 indicates major asymmetry, and (**b**) linear regression plot to assess publication bias in studies evaluating *Babesia* in dogs (Colored circles represent each study)
