# Supplementary material for: A global systematic review and meta‐analysis on the babesiosis in dogs with special reference to Babesia canis
Source: Vet Med Sci. 2024 May 2;10(3):e1427. doi: 10.1002/vms3.1427 (PMC11063922; doi:10.1002/vms3.1427)
Supplement: Supplementary file 10 — Supporting information [file VMS3-10-e1427-s003.docx]

Supplementary Table 1. Main characteristics of the included studies reporting the prevalence of *Babesia*.

| **Study No.** | **Author** | **Year** | **Country name** | **Continent** | **Time of sampling** | **Sample size** | **Infected** | **Type of *Babesia*** |
| --- | --- | --- | --- | --- | --- | --- | --- | --- |
| 1 | Ashrafi, et al. | 2001 | Iran | Asia | 1996–1999 | 281 | 1 | *Babesia* spp*.* |
| 2 | O’dwyer, et al. | 2001 | Brazil | South America | 1997–1998 | 250 | 13 | *Babesia* spp*.* |
| 3 | Pavlović, et al. | 2002 | Serbia | Europe | 1997–2001 | 3945 | 2922 | *Babesia* spp*.* |
| 4 | Rodrigues, et al. | 2002 | Brazil | South America | NS | 104 | 28 | *Babesia* spp*.* |
| 5 | Macintire, et al. | 2002 | U.S.A | North America | NS | 120 | 18 | *Babesia gibsoni* |
| 6 | Birkenheuer, et al. | 2003 | U.S.A | North America | NS | 508 | 43 | *Babesia gibsoni* |
| 7 | Jefferies, et al. | 2003 | Australia | Oceania | NS | 64 | 12 | *Babesia* spp*.* |
| 8 | Devalgas e bastos, et al. | 2004 | Brazil | South America | 1998–2001 | 194 | 61 | *Babesia* spp*.* |
| 9 | Inokuma, et al. | 2004 | Japan | Asia | 2001 | 80 | 12 | *Babesia canis*  *Babesia gibsoni* |
| 10 | Song, et al. | 2004 | South Korea | Asia | 2001–2002 | 501 | 9 | *Babesia gibsoni* |
| 11 | Matsuu, et al. | 2004 | Japan | Asia | 2002–2003 | 141 | 42 | *Babesia gibsoni* |
| 12 | Duh, et al. | 2004 | Slovenia | Asia | 2000–2002 | 476 | 28 | *Babesia* spp*.* |
| 13 | Matjila, et al. | 2004 | South Africa | Africa | NS | 297 | 44 | *Babesia* spp*.* |
| 14 | Ikadai, et al. | 2004 | Japan | Asia | 2002– 2003 | 1082 | 52 | *Babesia gibsoni* |
| 15 | Verdida, et al | 2004 | Japan | Asia | NS | 219 | 18 | *Babesia gibsoni* |
| 16 | Verdida, et al | 2004 | China | Asia | NS | 41 | 2 | *Babesia gibsoni* |
| 17 | Camacho, et al. | 2005 | Spain | Europe | 2001–2002 | 2979 | 87 | *Babesia* spp*.* |
| 18 | Oyamada, et al. | 2005 | Sudan | Africa | 1998–2000 | 78 | 7 | *Babesia rossi* |
| 19 | Birkenheuer, et al. | 2005 | U.S.A | North America | 2000–2003 | 673 | 144 | *Babesia gibsoni*  *Babesia vogeli* |
| 20 | Miyama, et al. | 2005 | Japan | Asia | NS | 230 | 56 | *Babesia gibsoni* |
| 21 | Gary, et al. | 2006 | Canada | North America | 2000–2003 | 288 | 58 | *Babesia* spp*.*  *Babesia gibsoni* |
| 22 | Brown, et al. | 2006 | Australia | Oceania | 2000–2004 | 215 | 22 | *Babesia* spp*.* |
| 23 | Hornok, et al. | 2006 | Hungary | Europe | 2005 | 651 | 37 | *Babesia* spp*.* |
| 24 | Soares, et al. | 2006 | Brazil | South America | 2003 | 101 | 2 | *Babesia* spp*.* |
| 25 | Trapp, et al. | 2006 | Brazil | South America | 2000–2001 | 381 | 136 | *Babesia vogeli* |
| 26 | Criado, et al. | 2006 | Spain | Europe | NS | 100 | 21 | *Babesia canis* |
| 27 | Criado-Fornelio, et al. | 2007 | Venezuela | South America | NS | 134 | 3 | *Babesia* spp*.* |
| 28 | Criado-Fornelio, et al. | 2007 | Spain | Europe | NS | 250 | 3 | *Babesia* spp*.* |
| 29 | Omudu, et al. | 2007 | Nigeria | Africa | NS | 108 | 11 | *Babesia* spp*.* |
| 30 | Jefferies, et al. | 2007 | Australia | Oceania | NS | 302 | 28 | *Babesia gibsoni* |
| 31 | Maia, et al. | 2007 | Brazil | South America | NS | 505 | 95 | *Babesia vogeli* |
| 32 | Sasaki, et al. | 2007 | Nigeria | Africa | NS | 400 | 9 | *Babesia* spp*.* |
| 33 | Cruz-Flores, et al. | 2008 | Brazil | South America | NS | 48 | 13 | *Babesia gibsoni* |
| 34 | Konishi, et al. | 2008 | Japan | Asia | 2005–2006 | 1206 | 128 | *Babesia gibsoni* |
| 35 | M’ghirbi and Bouattour. | 2008 | Tunisia | Africa | 2006 | 180 | 12 | *Babesia canis* |
| 36 | Matjila, et al. | 2008 | South Africa | Africa | 2000–2006 | 1138 | 438 | *Babesia vogeli*  *Babesia rossi* |
| 37 | Egege, et al. | 2008 | Nigeria | Africa | 1990–1999 | 180 | 97 | *Babesia* spp*.* |
| 38 | Solano-Gallego, et al. | 2008 | Italy | Europe | 2003–2008 | 164 | 45 | *Babesia canis*  *Babesia vogeli* |
| 39 | Yabsley, et al. | 2008 | Grenada | North America | 2006 | 73 | 5 | *Babesia vogeli* |
| 40 | Miranda, et al. | 2008 | Brazil | South America | NS | 2031 | 30 | *Babesia* spp*.* |
| 41 | Beck, et al. | 2009 | Croatia | Europe | 2007–2008 | 929 | 110 | *Babesia canis*  *Babesia vogeli*  *Babesia gibsoni*  *Babesia caballi*  *Babesia* spp*.*  *Babesia vulpes* |
| 42 | Cassini, et al. | 2009 | Italy | Europe | 2005–2006 | 1177 | 147 | *Babesia canis* |
| 43 | Bashir, et al. | 2009 | Pakistan | Asia | 2006 | 6204 | 163 | *Babesia* spp*.* |
| 44 | Costa-Júnior, et al. | 2009 | Brazil | South America | 2004 | 244 | 70 | *Babesia vogeli* |
| 45 | Criado-Fornelio, et al. | 2009 | France | Europe | 2006–2007 | 108 | 1 | *Babesia vogeli* |
| 46 | Furuta, et al. | 2009 | Brazil | South America | NS | 492 | 313 | *Babesia* spp*.* |
| 47 | Götsch, et al. | 2009 | Cape Verde | Africa | 2008 | 130 | 3 | *Babesia* spp*.* |
| 48 | Guimarães, et al. | 2009 | Brazil | South America | 2000–2002 | 300 | 220 | *Babesia* spp*.* |
| 49 | O'Dwyer, et al. | 2009 | Brazil | South America | 2004–2005 | 300 | 15 | *Babesia* spp*.* |
| 50 | Tabar, et al. | 2009 | Spain | Europe | 2005–2006 | 153 | 9 | *Babesia canis*  *Babesia vogeli*  *Babesia gibsoni*  *Babesia vulpes* |
| 51 | Welc-Falęciak, et al. | 2009 | Poland | Europe | 2006–2008 | 82 | 23 | *Babesia* spp*.* |
| 52 | Zygner, et al. | 2009 | Poland | Europe | 2003–2004 | 408 | 48 | *Babesia* spp*.* |
| 53 | Yeagley, et al. | 2009 | U.S.A | North America | 2006–2007 | 157 | 53 | *Babesia gibsoni* |
| 54 | Wu, et al. | 2009 | Taiwan | Asia | 2008–2008 | 101 | 7 | *Babesia gibsoni* |
| 55 | Spolidorio, et al. | 2010 | Brazil | South America | 200 –2008 | 92 | 20 | *Babesia* spp*.* |
| 56 | Lee, et al. | 2010 | Taiwan | Asia | 2008–2009 | 125 | 29 | *Babesia gibsoni* |
| 57 | de Almeida Curi, et al. | 2010 | Brazil | South America | 2004–2005 | 70 | 30 | *Babesia* spp*.* |
| 58 | Fritz. | 2010 | France | Europe | 2006–2008 | 166 | 108 | *Babesia canis*  *Babesia rossi*  *Babesia gibsoni*  *Babesia caballi* |
| 59 | Otranto, et al | 2010 | Italy | Europe | 2008 –2009 | 109 | 17 | *Babesia* spp*.* |
| 60 | Ramos, et al. | 2010 | Brazil | South America | 2007–2008 | 205 | 7 | *Babesia vogeli* |
| 61 | Wang, et al. | 2010 | U.S.A | North America | 2006–2009 | 235 | 25 | *Babesia gibsoni*  *Babesia vogeli* |
| 62 | Amuta, et al. | 2010 | Nigeria | Africa | NS | 108 | 11 | *Babesia* spp*.* |
| 63 | Cardoso, et al. | 2010 | Portugal | Europe | 2007–2009 | 45 | 36 | *Babesia canis* |
| 64 | Adaszek, et al. | 2011 | Poland | Europe | 2008–2010 | 800 | 158 | *Babesia* spp*.* |
| 65 | Jumde, et al. | 2011 | India | Asia | 2008–2008 | 150 | 12 | *Babesia* spp*.* |
| 66 | Kamani, et al. | 2011 | Nigeria | Africa | 2008– 2010 | 114 | 40 | *Babesia* spp*.* |
| 67 | Levy, et al. | 2011 | U.S.A | North America | 2005 | 342 | 7 | *Babesia gibsoni* |
| 68 | Abd Rani, et al. | 2011 | India | Asia | NS | 525 | 10 | *Babesia gibsoni*  *Babesia vogeli* |
| 69 | Varanat, et al. | 2011 | U.S.A | North America | NS | 137 | 3 | *Babesia* spp*.*  *Babesia gibsoni* |
| 70 | Majlathova, et al. | 2011 | Slovakia | Europe | 2004-2010 | 87 | 79 | *Babesia canis* |
| 71 | Bigdeli, et al. | 2012 | Iran | Asia | 2009–2011 | 280 | 1 | *Babesia* spp*.* |
| 72 | Buddhachat, et al. | 2012 | Thailand | Asia | 2008 | 102 | 23 | *Babesia vogeli* |
| 73 | Costa-Júnior, et al. | 2012 | Brazil | South America | 2004 | 418 | 43 | *Babesia vogeli* |
| 74 | Singh, et al. | 2012 | India | Asia | 2010 | 460 | 41 | *Babesia canis*  *Babesia gibsoni* |
| 75 | Hii, et al. | 2012 | Australia | Oceania | 2010 | 230 | 13 | *Babesia vogeli* |
| 76 | Konvalinová, et al. | 2012 | Czech Republic | Europe | 2010 | 68 | 6 | *Babesia* spp*.* |
| 77 | Pennisi, et al. | 2012 | Italy | Europe | 2009 | 249 | 45 | *Babesia* spp*.* |
| 78 | Vargas-Hernández, et al. | 2012 | Colombia | South America | NS | 182 | 52 | *Babesia vogeli* |
| 79 | Adamu, et al. | 2012 | Nigeria | Africa | 2010 | 181 | 5 | *Babesia* spp*.* |
| 80 | Kubelová, et al. | 2013 | Slovakia | Europe | 2010 | 584 | 43 | *Babesia* spp*.* |
| 81 | Imre, et al. | 2013 | Romania | Europe | 2011–2012 | 49 | 45 | *Babesia* spp*.* |
| 82 | Kamani, et al. | 2013 | Nigeria | Africa | 2011 | 181 | 6 | *Babesia rossi* |
| 83 | Kelly, et al. | 2013 | Saint Kitts and Nevis | North America | 2009–2011 | 372 | 48 | *Babesia vogeli*  *Babesia gibsoni* |
| 84 | Loftis, et al. | 2013 | Saint Kitts and Nevis | North America | 2009–2011 | 165 | 25 | *Babesia vogeli*  *Babesia gibsoni* |
| 85 | Okubanjo, et al. | 2013 | Nigeria | Africa | 2010 | 150 | 10 | *Babesia* spp*.* |
| 86 | Nwoha, et al. | 2013 | Nigeria | Africa | 2012 | 503 | 339 | *Babesia* spp*.* |
| 87 | Pam, et al. | 2013 | Nigeria | Africa | NS | 100 | 43 | *Babesia* spp*.* |
| 88 | Abdel-Rhman, et al. | 2014 | Egypt | Africa | 2014–2015 | 200 | 17 | *Babesia* spp*.* |
| 89 | Adamu, et al. | 2014 | Nigeria | Africa | 2010 | 100 | 31 | *Babesia rossi* |
| 90 | Jegede, et al. | 2014 | Nigeria | Africa | 2013–2014 | 101 | 9 | *Babesia* spp*.* |
| 91 | Laha, et al. | 2014 | India | Asia | NS | 222 | 56 | *Babesia gibsoni*  *Babesia* spp*.* |
| 92 | Moraes, et al. | 2014 | Brazil | South America | 2011 | 100 | 22 | *Babesia* spp*.* |
| 93 | Paulauskas, et al. | 2014 | Lithuania | Europe | 2013–2014 | 246 | 202 | *Babesia* spp*.* |
| 94 | Rojas, et al. | 2014 | Costa Rica | North America | 2012 | 146 | 12 | *Babesia vogeli* |
| 95 | Singh, et al. | 2014 | India | Asia | 2012–2013 | 428 | 49 | *Babesia gibsoni* |
| 96 | Minervino, et al. | 2015 | Brazil | South America | NS | 327 | 46 | *Babesia vogeli* |
| 97 | Cao, et al. | 2015 | China | Asia | 2013–2014 | 1170 | 108 | *Babesia gibsoni* |
| 98 | Terao, et al. | 2015 | Bangladesh | Asia | 2012 | 50 | 15 | *Babesia gibsoni* |
| 99 | Xu, et al. | 2015 | China | Asia | 2012–2014 | 1114 | 13 | *Babesia gibsoni*  *Babesia vogeli* |
| 100 | Aktas, et al. | 2015 | Turkey | Asia | 2010–2012 | 757 | 1 | *Babesia* spp*.* |
| 101 | Araujo, et al. | 2015 | Brazil | South America | 2011–2012 | 404 | 234 | *Babesia vogeli* |
| 102 | Das, et al. | 2015 | India | Asia | 2012–2013 | 226 | 72 | *Babesia* spp*.* |
| 103 | Davitkov, et al. | 2015 | Serbia | Europe | 2012–2014 | 120 | 118 | *Babesia gibsoni*  *Babesia* spp*.* |
| 104 | El-Dakhly, et al. | 2015 | Japan | Asia | 2011 | 392 | 87 | *Babesia gibsoni* |
| 105 | Gabrielli, et al. | 2015 | Serbia | Europe | 2012–2014 | 158 | 34 | *Babesia vogeli*  *Babesia vulpes*  *Babesia gibsoni*  *Babesia caballi*  *Babesia* spp*.* |
| 106 | Krawczak, et al. | 2015 | Brazil | South America | 2013 | 96 | 30 | *Babesia vogeli* |
| 107 | Kubo, et al. | 2015 | Japan | Asia | 2012–2013 | 722 | 17 | *Babesia gibsoni*  *Babesia* spp*.* |
| 108 | Mbugua, et al. | 2015 | Kenya | Africa | 1987–2010 | 88104 | 1248 | *Babesia* spp*.* |
| 109 | Miró, et al. | 2015 | Spain | Europe | 2012–2014 | 240 | 112 | *Babesia vulpes* |
| 110 | Pantchev, et al. | 2015 | Bulgaria | Europe | NS | 167 | 27 | *Babesia* spp*.* |
| 111 | Piratae, et al. | 2015 | Thailand | Asia | 2014 | 79 | 5 | *Babesia vogeli* |
| 112 | René-Martellet, et al. | 2015 | France | Europe | 2010–2012 | 140 | 37 | *Babesia* spp*.*  *Babesia vogeli* |
| 113 | Vipan, et al. | 2015 | India | Asia | 2013–2014 | 204 | 17 | *Babesia gibsoni*  *Babesia* spp*.* |
| 114 | Giudice, et al. | 2015 | Italy | Europe | 2005 | 86 | 1 | *Babesia vulpes* |
| 115 | Hosseinzadeh Varjoy, et al. | 2016 | Iran | Asia | NS | 242 | 30 | *Babesia* spp*.* |
| 116 | Singh, et al. | 2016 | India | Asia | 2014-2015 | 164 | 13 | *Babesia gibsoni* |
| 117 | Bhaskaran Ravi, et al. | 2016 | India | Asia | 2006–2013 | 28343 | 1288 | *Babesia gibsoni*  *Babesia* spp*.* |
| 118 | Sudhakara Reddy, et al | 2016 | India | Asia | 2012 –2013 | 300 | 6 | *Babesia* spp*.* |
| 119 | Akhtardanesh, et al. | 2016 | Iran | Asia | 2012–2014 | 60 | 3 | *Babesia gibsoni* |
| 120 | da Silva, et al. | 2016 | Brazil | South America | NS | 146 | 7 | *Babesia vogeli* |
| 121 | Hamel, et al. | 2016 | Albania | Europe | 2010–2011 | 602 | 18 | *Babesia gibsoni* |
| 122 | Liu, et al. | 2016 | Thailand | Asia | NS | 181 | 5 | *Babesia* spp*.* |
| 123 | Mascarelli, et al. | 2016 | Argentina | South America | NS | 70 | 3 | *Babesia vogeli* |
| 124 | Rjeibi, et al. | 2016 | Tunisia | Africa | 2014 | 200 | 25 | *Babesia vogeli* |
| 125 | Singla, et al. | 2016 | India | Asia | 2014 | 778 | 119 | *Babesia gibsoni*  *Babesia vogeli* |
| 126 | Starkey, et al. | 2016 | Haiti | North America | 2013 | 207 | 8 | *Babesia vogeli* |
| 127 | Mrljak, et al. | 2017 | Croatia | Europe | NS | 435 | 75 | *Babesia* spp*.* |
| 128 | Zheng, et al. | 2017 | China | Asia | NS | 162 | 12 | *Babesia gibsoni*  *Babesia vogeli* |
| 129 | Adao, et al. | 2017 | Philippines | Asia | 2013–2014 | 114 | 6 | *Babesia vogeli* |
| 130 | Akande, et al. | 2017 | Nigeria | Africa | 2014–2015 | 218 | 76 | *Babesia* spp*.* |
| 131 | Aktas and Ozubek. | 2017 | Turkey | Asia | 2015 | 219 | 7 | *Babesia* spp*.*  *Babesia vogeli*  *Babesia gibsoni* |
| 132 | Andersson, et al. | 2017 | Romania | Europe | 2013– 2014 | 96 | 29 | *Babesia gibsoni*  *Babesia* spp*.* |
| 133 | Augustine, al. | 2017 | India | Asia | NS | 160 | 72 | *Babesia gibsoni*  *Babesia vogeli*  *Babesia* spp*.* |
| 134 | Azmi, al. | 2017 | Palestine | Asia | 2010–2015 | 362 | 9 | *Babesia* spp*.*  *Babesia vogeli* |
| 135 | Figueredo, al. | 2017 | Brazil | South America | 2015 | 300 | 51 | *Babesia* spp. |
| 136 | Guven, al. | 2017 | Turkey | Asia | 2012–2013 | 133 | 7 | *Babesia* spp*.* |
| 137 | Harvey, al. | 2017 | Brazil | South America | NS | 760 | 287 | *Babesia* spp*.* |
| 138 | Kebede and Dereje. | 2017 | Ethiopia | Africa | 2014–2015 | 384 | 61 | *Babesia gibsoni*  *Babesia* spp*.* |
| 139 | Mohammed, et al. | 2017 | Malaysia | Asia | 2013–2014 | 104 | 68 | *Babesia* spp*.* |
| 140 | Ribeiro, et al. | 2017 | Brazil | South America | 2014 | 182 | 20 | *Babesia vogeli* |
| 141 | Zhang, et al. | 2017 | China | Asia | 2013 | 562 | 22 | *Babesia gibsoni* |
| 142 | Bigdeli and Namavari. | 2017 | Iran | Asia | NS | 280 | 3 | *Babesia* spp*.* |
| 143 | Jikuya, et al | 2017 | Japan | Asia | 2012–2015 | 88 | 2 | *Babesia gibsoni* |
| 144 | Niu, et al. | 2017 | China | Asia | 2015–2016 | 141 | 2 | *Babesia vogeli* |
| 145 | Pavlović, et al. | 2017 | Serbia | Europe | 2014–2015 | 249 | 147 | *Babesia gibsoni*  *Babesia* spp*.* |
| 146 | Annoscia, et al. | 2017 | Italy | Europe | NS | 147 | 15 | *Babesia vogeli*  *Babesia canis* |
| 147 | He, et al. | 2017 | China | Asia | NS | 236 | 24 | *Babesia gibsoni* |
| 148 | Ahmad, et al. | 2018 | Pakistan | Asia | 2016 | 900 | 302 | *Babesia gibsoni*  *Babesia vogeli* |
| 149 | Azhahianambi, et al. | 2018 | India | Asia | 2014–2017 | 287 | 89 | *Babesia* spp*.* |
| 150 | David, et al. | 2018 | Nigeria | Africa | 2012 | 129 | 30 | *Babesia* spp*.* |
| 151 | Ybañez, et al. | 2018 | Philippines | Asia | NS | 100 | 16 | *Babesia* spp*.* |
| 152 | Bano and Chandan. | 2018 | India | Asia | NS | 250 | 82 | *Babesia* spp*.* |
| 153 | Ehimiyein, et al. | 2018 | Nigeria | Africa | 2015 | 61 | 10 | *Babesia* spp*.* |
| 154 | Filipović, et al. | 2018 | Serbia | Europe | 2015 | 111 | 18 | *Babesia* spp*.*  *Babesia gibsoni* |
| 155 | Happi, et al. | 2018 | Nigeria | Africa | 2013 | 232 | 41 | *Babesia* spp*.* |
| 156 | Singh, et al. | 2018 | India | Asia | 2015–2016 | 5711 | 11 | *Babesia* spp*.* |
| 157 | Kirade, et al. | 2018 | India | Asia | 2016–2017 | 150 | 12 | *Babesia* spp*.*  *Babesia gibsoni* |
| 158 | Kushwaha, et al. | 2018 | India | Asia | 2014–2015 | 14322 | 237 | *Babesia gibsoni* |
| 159 | Jain, et al. | 2018 | India | Asia | NS | 300 | 95 | *Babesia gibsoni*  *Babesia vogeli* |
| 160 | de Toledo Vieira, et al. | 2018 | Brazil | South America | NS | 378 | 5 | *Babesia* spp*.* |
| 161 | Temoche, et al. | 2018 | Peru | South America | 2014–2015 | 212 | 3 | *Babesia vogeli* |
| 162 | Ćoralić, et al. | 2018 | Bosnia and Herzegovina | Europe | 2014–2016 | 80 | 66 | *Babesia canis* |
| 163 | Barash, et al. | 2019 | U.S.A | North America | 2015–2018 | 9367 | 219 | *Babesia gibsoni*  *Babesia vogeli*  *Babesia* spp*.*  *Babesia vulpes*  *Babesia conradae*  *Babesia canis* |
| 164 | Wang, et al. | 2019 | China | Asia | 2017–2018 | 130 | 7 | *Babesia canis* |
| 165 | Betgiri, et al. | 2019 | India | Asia | NS | 150 | 66 | *Babesia gibsoni* |
| 166 | Tayyub, et al. | 2019 | Pakistan | Asia | NS | 200 | 84 | *Babesia* spp*.* |
| 167 | Inácio, et al. | 2019 | Paraguay | South America | NS | 384 | 21 | *Babesia vogeli* |
| 168 | Divya, et al. | 2019 | India | Asia | 2018–2019 | 2345 | 340 | *Babesia gibsoni*  *Babesia* spp*.* |
| 169 | Akram, et al. | 2019 | Pakistan | Asia | NS | 150 | 2 | *Babesia vulpes* |
| 170 | Bilgic, et al. | 2019 | Turkey | Asia | 2004 | 379 | 8 | *Babesia vogeli* |
| 171 | Braga, et al. | 2019 | Brazil | South America | NS | 945 | 175 | *Babesia vogeli* |
| 172 | Checa, et al. | 2019 | Spain | Europe | 2017–2018 | 1512 | 155 | *Babesia vulpes*  *Babesia* spp*.* |
| 173 | Modarelli, et al. | 2019 | U.S.A | North America | 2016–2018 | 1171 | 5 | *Babesia gibsoni* |
| 174 | Springer, et al. | 2019 | Costa Rica | North America | 2014 | 294 | 9 | *Babesia* spp*.* |
| 175 | Dhliwayo, et al. | 2019 | Zimbabwe | Africa | 2016–2017 | 117 | 56 | *Babesia* spp*.* |
| 176 | Leica, et al. | 2019 | Romania | Europe | 2016–2018 | 306 | 85 | *Babesia* spp*.* |
| 177 | Zuchi, et al. | 2020 | Brazil | South America | NS | 424 | 178 | *Babesia* spp*.* |
| 178 | Teodorowski, et al. | 2020 | Poland | Europe | 2018–2020 | 216 | 102 | *Babesia gibsoni*  *Babesia* spp*.* |
| 179 | Hassanen. | 2020 | Egypt | Africa | 2018–2019 | 150 | 23 | *Babesia vogeli* |
| 180 | Mehta, et al. | 2020 | India | Asia | 2018–2019 | 3500 | 36 | *Babesia* spp*.* |
| 181 | Bahiense, et al. | 2020 | Brazil | South America | 2013–2015 | 175 | 6 | *Babesia vogeli* |
| 182 | Alanazi, et al. | 2020 | Saudi Arabia | Asia | 2018–2019 | 70 | 21 | *Babesia vogeli* |
| 183 | Ngoka, et al. | 2020 | Kenya | Africa | NS | 143 | 13 | *Babesia vogeli*  *Babesia rossi* |
| 184 | Paschoal, et al. | 2020 | Brazil | South America | 2014–2016 | 461 | 49 | *Babesia vogeli* |
| 185 | Medkour, et al. | 2020 | Côte d’Ivoire | Africa | NS | 123 | 1 | *Babesia vogeli* |
| 186 | Badawi and Yousif. | 2020 | Iraq | Asia | 2018–2019 | 620 | 21 | *Babesia canis*  *Babesia vogeli*  *Babesia rossi* |
| 187 | Castro, et al. | 2020 | Brazil | South America | NS | 407 | 10 | *Babesia vogeli* |
| 188 | Cimpan, et al. | 2020 | Romania | Europe | 2017 | 300 | 20 | *Babesia* spp*.* |
| 189 | Guo, et al. | 2020 | China | Asia | 2017–2018 | 371 | 175 | *Babesia gibsoni* |
| 190 | Kaur, et al. | 2020 | India | Asia | 2018–2019 | 644 | 4 | *Babesia vogeli* |
| 191 | Kiouani, et al. | 2020 | Algeria | Africa | 2015–2016 | 378 | 59 | *Babesia* spp*.*  *Babesia gibsoni* |
| 192 | Lee, et al. | 2020 | Korea | Asia | 2017–2018 | 2215 | 38 | *Babesia gibsoni* |
| 193 | Manoj, et al. | 2020 | India | Asia | 2018 | 230 | 24 | *Babesia gibsoni*  *Babesia vogeli* |
| 194 | Obeta, et al. | 2020 | Nigeria | Africa | 2015–2016 | 480 | 52 | *Babesia vogeli* |
| 195 | Seleznova, et al. | 2020 | Latvia | Europe | 2016–2019 | 524 | 62 | *Babesia* spp*.* |
| 196 | Senthil, et al. | 2020 | India | Asia | 2010 to 2019 | 11000 | 1187 | *Babesia* spp*.*  *Babesia gibsoni* |
| 197 | Thomas, et al. | 2020 | Colombia | South America | 2017 | 169 | 15 | *Babesia* spp*.* |
| 198 | Wang, et al. | 2020 | China | Asia | NS | 115 | 5 | *Babesia* spp*.*  *Babesia vogeli* |
| 199 | Zanet, et al. | 2020 | Italy | Europe | NS | 1454 | 323 | *Babesia* spp*.*  *Babesia vogeli*  *Babesia vulpes* |
| 200 | Li, et al. | 2020 | China | Asia | 2018 | 272 | 30 | *Babesia vogeli* |
| 201 | ASLANTAŞ, et al. | 2020 | Turkey | Asia | 2020 | 186 | 2 | *Babesia vogeli* |
| 202 | Díaz-Regañón, et al. | 2020 | Nepal | Africa | 2017 | 70 | 2 | *Babesia vogeli* |
| 203 | Habibi, et al. | 2020 | Iran | Asia | 2016–2017 | 40 | 10 | *Babesia vogeli* |
| 204 | Abdullah, et al. | 2021 | Egypt | Africa | 2016–2018 | 203 | 1 | *Babesia* spp*.* |
| 205 | Zaki, et al. | 2021 | Egypt | Africa | 2017–2019 | 242 | 62 | *Babesia vogeli* |
| 206 | Bouattour, et al. | 2021 | Tunisia | Africa | 2018 | 99 | 3 | *Babesia vogeli* |
| 207 | Khanmohammadi, et al. | 2021 | Iran | Asia | 2017–2018 | 43 | 4 | *Babesia* spp*.* |
| 208 | Kopparthi, et al. | 2021 | India | Asia | 2019–2020 | 884 | 177 | *Babesia gibsoni*  *Babesia vogeli* |
| 209 | Preena, et al. | 2021 | India | Asia | 2018–2020 | 4039 | 35 | *Babesia* spp*.* |
| 210 | Ngoka, et al. | 2021 | Kenya | Africa | 2018–2019 | 143 | 13 | *Babesia vogeli*  *Babesia rossi,* |
| 211 | Nhuong, et al. | 2021 | Cambodia | Asia | 2014–2015 | 455 | 1 | *Babesia vogeli* |
| 212 | García-Quesada, et al. | 2021 | Costa Rica | North America | 2011–2014 | 853 | 149 | *Babesia vogeli*  *Babesia* spp*.* |
| 213 | Zeng, et al. | 2021 | China | Asia | 2015–2020 | 56 | 1 | *Babesia* spp*.* |
| 214 | Stayton, et al. | 2021 | U.S.A | North America | NS | 40 | 15 | *Babesia conradae* |
| 215 | Carli, et al. | 2021 | Italy | Europe | 2016 - 2019 | 607 | 40 | *Babesia vogeli*  *Babesia gibsoni*  *Babesia canis*  *Babesia vulpes* |
| 216 | Bawm, et al. | 2021 | Myanmar | Africa | 2013 - 2016 | 91 | 7 | *Babesia vogeli*  *Babesia gibsoni* |
| 217 | Panda, et al. | 2022 | India | Asia | NS | 198 | 38 | *Babesia vogeli* |
| 218 | Dordio, et al. | 2022 | Portugal | Europe | 2016– 2017 | 142 | 6 | *Babesia vogeli*  *Babesia* spp*.* |
| 219 | Ikejiofor, et al. | 2021 | Nigeria | Africa | NS | 150 | 16 | *Babesia* spp*.* |
| 220 | Morelli, et al. | 2021 | Italy | Europe | NS | 294 | 25 | *Babesia* spp*.* |
| 221 | Dos Santos, et al | 2022 | Brazil | South America | 2018–2019 | 407 | 33 | *Babesia vogeli* |
| 222 | AbdElmaged. | 2022 | Egypt | Africa | 2021 | 80 | 12 | *Babesia* spp*.* |
| 223 | Bordoloi, et al | 2022 | India | Asia | 2017–2019 | 205 | 68 | *Babesia gibsoni*  *Babesia* spp*.* |
| 224 | Fonsêca, et al | 2022 | Brazil | South America | 2019–2020 | 153 | 19 | *Babesia vogeli* |
| 225 | Niestat, et al | 2022 | U.S.A | North America | 2011–2019 | 1709 | 286 | *Babesia gibsoni* |
| 226 | Padmaja, et al | 2022 | India | Asia | NS | 400 | 22 | *Babesia vogeli*  *Babesia gibsoni* |
| 227 | Yang, et al | 2022 | Taiwan | Asia | 2019–2020 | 530 | 67 | *Babesia vogeli*  *Babesia gibsoni* |
| 228 | Aitor, et al. | 2022 | Chile | South America | NS | 764 | 5 | *Babesia vogeli* |
| 229 | Zeng, et al. | 2022 | China | Asia | 2015–2020 | 56 | 1 | *Babesia* spp*.* |
