# Supplementary material for: A global systematic review and meta‐analysis on the babesiosis in dogs with special reference to Babesia canis
Source: Vet Med Sci. 2024 May 2;10(3):e1427. doi: 10.1002/vms3.1427 (PMC11063922; doi:10.1002/vms3.1427)
Supplement: Supplementary file 11 — Supporting information [file VMS3-10-e1427-s002.docx]

Supplementary Table 2. Main characteristics of the included studies reporting the prevalence of *Babesia*.

| **Study No.** | **Author** | **Year** | **Country name** | **Selection**  **(maximum of 5 stars)** | **Comparability**  **(maximum of 2 stars)** | **Outcome**  **(maximum of 3 stars)** | **Total Score** |
| --- | --- | --- | --- | --- | --- | --- | --- |
| 1 | Ashrafi, et al. | 2001 | Iran | **** | ** | ** | 8 |
| 2 | O’dwyer, et al. | 2001 | Brazil | *** | * | ** | 6 |
| 3 | Pavlović, et al. | 2002 | Serbia | *** | * | ** | 6 |
| 4 | Rodrigues, et al. | 2002 | Brazil | *** | * | *** | 7 |
| 5 | Macintire, et al. | 2002 | U.S.A | *** | ** | *** | 8 |
| 6 | Birkenheuer, et al. | 2003 | U.S.A | **** | ** | ** | 8 |
| 7 | Jefferies, et al. | 2003 | Australia | *** | * | ** | 6 |
| 8 | Devalgas e bastos, et al. | 2004 | Brazil | ** | * | ** | 5 |
| 9 | Inokuma, et al. | 2004 | Japan | *** | * | *** | 7 |
| 10 | Song, et al. | 2004 | South Korea | ** | * | ** | 5 |
| 11 | Matsuu, et al. | 2004 | Japan | *** | ** | *** | 8 |
| 12 | Duh, et al. | 2004 | Slovenia | *** | * | ** | 6 |
| 13 | Matjila, et al. | 2004 | South Africa | *** | ** | *** | 8 |
| 14 | Ikadai, et al. | 2004 | Japan | *** | * | *** | 7 |
| 15 | Verdida, et al | 2004 | Japan | **** | ** | ** | 8 |
| 16 | Verdida, et al | 2004 | China | *** | * | ** | 6 |
| 17 | Camacho, et al. | 2005 | Spain | ** | * | ** | 5 |
| 18 | Oyamada, et al. | 2005 | Sudan | *** | * | *** | 7 |
| 19 | Birkenheuer, et al. | 2005 | U.S.A | ** | * | ** | 5 |
| 20 | Miyama, et al. | 2005 | Japan | *** | ** | *** | 8 |
| 21 | Gary, et al. | 2006 | Canada | *** | * | ** | 6 |
| 22 | Brown, et al. | 2006 | Australia | *** | ** | *** | 8 |
| 23 | Hornok, et al. | 2006 | Hungary | *** | * | *** | 7 |
| 24 | Soares, et al. | 2006 | Brazil | ** | ** | *** | 7 |
| 25 | Trapp, et al. | 2006 | Brazil | *** | ** | ** | 7 |
| 26 | Criado, et al. | 2006 | Spain | **** | * | *** | 8 |
| 27 | Criado-Fornelio, et al. | 2007 | Venezuela | *** | * | *** | 7 |
| 28 | Criado-Fornelio, et al. | 2007 | Spain | *** | ** | *** | 8 |
| 29 | Omudu, et al. | 2007 | Nigeria | **** | ** | ** | 8 |
| 30 | Jefferies, et al. | 2007 | Australia | **** | * | ** | 7 |
| 31 | Maia, et al. | 2007 | Brazil | *** | ** | *** | 8 |
| 32 | Sasaki, et al. | 2007 | Nigeria | *** | ** | *** | 8 |
| 33 | Cruz-Flores, et al. | 2008 | Brazil | ** | ** | *** | 7 |
| 34 | Konishi, et al. | 2008 | Japan | *** | ** | ** | 7 |
| 35 | M’ghirbi and Bouattour. | 2008 | Tunisia | **** | * | *** | 8 |
| 36 | Matjila, et al. | 2008 | South Africa | *** | * | *** | 7 |
| 37 | Egege, et al. | 2008 | Nigeria | *** | ** | *** | 8 |
| 38 | Solano-Gallego, et al. | 2008 | Italy | ** | ** | ** | 6 |
| 39 | Yabsley, et al. | 2008 | Grenada | ** | * | ** | 5 |
| 40 | Miranda, et al. | 2008 | Brazil | *** | ** | *** | 8 |
| 41 | Beck, et al. | 2009 | Croatia | ** | ** | *** | 7 |
| 42 | Cassini, et al. | 2009 | Italy | *** | * | ** | 6 |
| 43 | Bashir, et al. | 2009 | Pakistan | **** | ** | *** | 9 |
| 44 | Costa-Júnior, et al. | 2009 | Brazil | *** | ** | ** | 7 |
| 45 | Criado-Fornelio, et al. | 2009 | France | ** | * | ** | 5 |
| 46 | Furuta, et al. | 2009 | Brazil | *** | * | *** | 7 |
| 47 | Götsch, et al. | 2009 | Cape Verde | *** | ** | *** | 8 |
| 48 | Guimarães, et al. | 2009 | Brazil | *** | ** | *** | 8 |
| 49 | O'Dwyer, et al. | 2009 | Brazil | ** | * | ** | 5 |
| 50 | Tabar, et al. | 2009 | Spain | *** | ** | *** | 8 |
| 51 | Welc-Falęciak, et al. | 2009 | Poland | **** | ** | *** | 9 |
| 52 | Zygner, et al. | 2009 | Poland | *** | * | ** | 6 |
| 53 | Yeagley, et al. | 2009 | U.S.A | *** | ** | *** | 8 |
| 54 | Wu, et al. | 2009 | Taiwan | *** | * | *** | 7 |
| 55 | Spolidorio, et al. | 2010 | Brazil | *** | ** | *** | 8 |
| 56 | Lee, et al. | 2010 | Taiwan | *** | ** | *** | 8 |
| 57 | de Almeida Curi, et al. | 2010 | Brazil | ** | * | ** | 5 |
| 58 | Fritz. | 2010 | France | *** | ** | *** | 8 |
| 59 | Otranto, et al | 2010 | Italy | **** | ** | *** | 9 |
| 60 | Ramos, et al. | 2010 | Brazil | *** | * | ** | 6 |
| 61 | Wang, et al. | 2010 | U.S.A | **** | ** | ** | 8 |
| 62 | Amuta, et al. | 2010 | Nigeria | **** | * | ** | 7 |
| 63 | Cardoso, et al. | 2010 | Portugal | ** | * | ** | 5 |
| 64 | Adaszek, et al. | 2011 | Poland | *** | ** | *** | 8 |
| 65 | Jumde, et al. | 2011 | India | ** | ** | *** | 7 |
| 66 | Kamani, et al. | 2011 | Nigeria | *** | ** | *** | 8 |
| 67 | Levy, et al. | 2011 | U.S.A | **** | ** | *** | 9 |
| 68 | Abd Rani, et al. | 2011 | India | *** | * | ** | 6 |
| 69 | Varanat, et al. | 2011 | U.S.A | **** | ** | ** | 8 |
| 70 | Majlathova, et al. | 2011 | Slovakia | **** | * | ** | 7 |
| 71 | Bigdeli, et al. | 2012 | Iran | *** | ** | ** | 7 |
| 72 | Buddhachat, et al. | 2012 | Thailand | *** | ** | *** | 8 |
| 73 | Costa-Júnior, et al. | 2012 | Brazil | ** | ** | *** | 7 |
| 74 | Singh, et al. | 2012 | India | **** | * | *** | 8 |
| 75 | Hii, et al. | 2012 | Australia | *** | * | *** | 7 |
| 76 | Konvalinová, et al. | 2012 | Czech Republic | ** | ** | ** | 6 |
| 77 | Pennisi, et al. | 2012 | Italy | *** | ** | *** | 8 |
| 78 | Vargas-Hernández, et al. | 2012 | Colombia | ** | ** | ** | 6 |
| 79 | Adamu, et al. | 2012 | Nigeria | **** | ** | ** | 8 |
| 80 | Kubelová, et al. | 2013 | Slovakia | *** | * | ** | 6 |
| 81 | Imre, et al. | 2013 | Romania | *** | * | ** | 6 |
| 82 | Kamani, et al. | 2013 | Nigeria | *** | * | *** | 7 |
| 83 | Kelly, et al. | 2013 | Saint Kitts and Nevis | *** | ** | *** | 8 |
| 84 | Loftis, et al. | 2013 | Saint Kitts and Nevis | *** | ** | *** | 8 |
| 85 | Okubanjo, et al. | 2013 | Nigeria | *** | ** | ** | 7 |
| 86 | Nwoha, et al. | 2013 | Nigeria | *** | ** | *** | 8 |
| 87 | Pam, et al. | 2013 | Nigeria | ** | ** | *** | 7 |
| 88 | Abdel-Rhman, et al. | 2014 | Egypt | **** | * | *** | 8 |
| 89 | Adamu, et al. | 2014 | Nigeria | *** | * | *** | 7 |
| 90 | Jegede, et al. | 2014 | Nigeria | ** | ** | ** | 6 |
| 91 | Laha, et al. | 2014 | India | *** | ** | *** | 8 |
| 92 | Moraes, et al. | 2014 | Brazil | ** | ** | ** | 6 |
| 93 | Paulauskas, et al. | 2014 | Lithuania | **** | ** | ** | 8 |
| 94 | Rojas, et al. | 2014 | Costa Rica | *** | * | *** | 7 |
| 95 | Singh, et al. | 2014 | India | *** | ** | *** | 8 |
| 96 | Minervino, et al. | 2015 | Brazil | ** | ** | ** | 6 |
| 97 | Cao, et al. | 2015 | China | *** | ** | ** | 7 |
| 98 | Terao, et al. | 2015 | Bangladesh | *** | ** | *** | 8 |
| 99 | Xu, et al. | 2015 | China | ** | ** | *** | 7 |
| 100 | Aktas, et al. | 2015 | Turkey | **** | * | *** | 8 |
| 101 | Araujo, et al. | 2015 | Brazil | *** | * | *** | 7 |
| 102 | Das, et al. | 2015 | India | ** | ** | ** | 6 |
| 103 | Davitkov, et al. | 2015 | Serbia | ** | ** | *** | 7 |
| 104 | El-Dakhly, et al. | 2015 | Japan | ** | ** | ** | 6 |
| 105 | Gabrielli, et al. | 2015 | Serbia | **** | ** | ** | 8 |
| 106 | Krawczak, et al. | 2015 | Brazil | *** | * | *** | 7 |
| 107 | Kubo, et al. | 2015 | Japan | *** | * | *** | 7 |
| 108 | Mbugua, et al. | 2015 | Kenya | ** | ** | *** | 7 |
| 109 | Miró, et al. | 2015 | Spain | ** | ** | *** | 7 |
| 110 | Pantchev, et al. | 2015 | Bulgaria | *** | ** | *** | 8 |
| 111 | Piratae, et al. | 2015 | Thailand | **** | ** | *** | 9 |
| 112 | René-Martellet, et al. | 2015 | France | *** | * | ** | 6 |
| 113 | Vipan, et al. | 2015 | India | **** | ** | ** | 8 |
| 114 | Giudice, et al. | 2015 | Italy | **** | * | ** | 7 |
| 115 | Hosseinzadeh Varjoy, et al. | 2016 | Iran | *** | ** | ** | 7 |
| 116 | Singh, et al. | 2016 | India | *** | ** | *** | 8 |
| 117 | Bhaskaran Ravi, et al. | 2016 | India | ** | ** | *** | 7 |
| 118 | Sudhakara Reddy, et al | 2016 | India | ** | ** | ** | 6 |
| 119 | Akhtardanesh, et al. | 2016 | Iran | *** | * | *** | 7 |
| 120 | da Silva, et al. | 2016 | Brazil | *** | ** | *** | 8 |
| 121 | Hamel, et al. | 2016 | Albania | *** | ** | *** | 8 |
| 122 | Liu, et al. | 2016 | Thailand | ** | ** | *** | 7 |
| 123 | Mascarelli, et al. | 2016 | Argentina | *** | ** | *** | 8 |
| 124 | Rjeibi, et al. | 2016 | Tunisia | *** | ** | *** | 8 |
| 125 | Singla, et al. | 2016 | India | ** | ** | *** | 7 |
| 126 | Starkey, et al. | 2016 | Haiti | ** | ** | ** | 6 |
| 127 | Mrljak, et al. | 2017 | Croatia | *** | * | *** | 7 |
| 128 | Zheng, et al. | 2017 | China | *** | ** | *** | 8 |
| 129 | Adao, et al. | 2017 | Philippines | *** | ** | *** | 8 |
| 130 | Akande, et al. | 2017 | Nigeria | ** | ** | *** | 7 |
| 131 | Aktas and Ozubek. | 2017 | Turkey | *** | ** | *** | 8 |
| 132 | Andersson, et al. | 2017 | Romania | **** | ** | *** | 9 |
| 133 | Augustine, al. | 2017 | India | *** | * | ** | 6 |
| 134 | Azmi, al. | 2017 | Palestine | ** | ** | *** | 7 |
| 135 | Figueredo, al. | 2017 | Brazil | *** | ** | *** | 8 |
| 136 | Guven, al. | 2017 | Turkey | ** | ** | *** | 7 |
| 137 | Harvey, al. | 2017 | Brazil | **** | * | *** | 8 |
| 138 | Kebede and Dereje. | 2017 | Ethiopia | *** | * | *** | 7 |
| 139 | Mohammed, et al. | 2017 | Malaysia | ** | ** | *** | 7 |
| 140 | Ribeiro, et al. | 2017 | Brazil | ** | ** | *** | 7 |
| 141 | Zhang, et al. | 2017 | China | *** | * | ** | 6 |
| 142 | Bigdeli and Namavari. | 2017 | Iran | *** | ** | *** | 8 |
| 143 | Jikuya, et al | 2017 | Japan | ** | ** | ** | 6 |
| 144 | Niu, et al. | 2017 | China | ** | ** | *** | 7 |
| 145 | Pavlović, et al. | 2017 | Serbia | ** | ** | *** | 7 |
| 146 | Annoscia, et al. | 2017 | Italy | ** | ** | *** | 7 |
| 147 | He, et al. | 2017 | China | ** | ** | ** | 6 |
| 148 | Ahmad, et al. | 2018 | Pakistan | **** | ** | ** | 8 |
| 149 | Azhahianambi, et al. | 2018 | India | *** | * | *** | 7 |
| 150 | David, et al. | 2018 | Nigeria | *** | * | *** | 7 |
| 151 | Ybañez, et al. | 2018 | Philippines | ** | ** | *** | 7 |
| 152 | Bano and Chandan. | 2018 | India | ** | ** | *** | 7 |
| 153 | Ehimiyein, et al. | 2018 | Nigeria | *** | * | *** | 7 |
| 154 | Filipović, et al. | 2018 | Serbia | ** | ** | *** | 7 |
| 155 | Happi, et al. | 2018 | Nigeria | ** | ** | *** | 7 |
| 156 | Singh, et al. | 2018 | India | *** | ** | *** | 8 |
| 157 | Kirade, et al. | 2018 | India | **** | ** | *** | 9 |
| 158 | Kushwaha, et al. | 2018 | India | *** | ** | ** | 7 |
| 159 | Jain, et al. | 2018 | India | *** | ** | *** | 8 |
| 160 | de Toledo Vieira, et al. | 2018 | Brazil | ** | ** | *** | 7 |
| 161 | Temoche, et al. | 2018 | Peru | ** | ** | ** | 6 |
| 162 | Ćoralić, et al. | 2018 | Bosnia and Herzegovina | *** | * | *** | 7 |
| 163 | Barash, et al. | 2019 | U.S.A | *** | ** | *** | 8 |
| 164 | Wang, et al. | 2019 | China | *** | ** | *** | 8 |
| 165 | Betgiri, et al. | 2019 | India | ** | ** | *** | 7 |
| 166 | Tayyub, et al. | 2019 | Pakistan | *** | ** | *** | 8 |
| 167 | Inácio, et al. | 2019 | Paraguay | *** | ** | *** | 8 |
| 168 | Divya, et al. | 2019 | India | ** | ** | *** | 7 |
| 169 | Akram, et al. | 2019 | Pakistan | ** | ** | ** | 6 |
| 170 | Bilgic, et al. | 2019 | Turkey | *** | * | *** | 7 |
| 171 | Braga, et al. | 2019 | Brazil | *** | ** | *** | 8 |
| 172 | Checa, et al. | 2019 | Spain | *** | ** | *** | 8 |
| 173 | Modarelli, et al. | 2019 | U.S.A | ** | ** | *** | 7 |
| 174 | Springer, et al. | 2019 | Costa Rica | *** | ** | *** | 8 |
| 175 | Dhliwayo, et al. | 2019 | Zimbabwe | **** | ** | *** | 9 |
| 176 | Leica, et al. | 2019 | Romania | *** | * | *** | 7 |
| 177 | Zuchi, et al. | 2020 | Brazil | ** | ** | *** | 7 |
| 178 | Teodorowski, et al. | 2020 | Poland | ** | ** | *** | 7 |
| 179 | Hassanen. | 2020 | Egypt | *** | ** | *** | 8 |
| 180 | Mehta, et al. | 2020 | India | **** | ** | *** | 9 |
| 181 | Bahiense, et al. | 2020 | Brazil | *** | * | ** | 6 |
| 182 | Alanazi, et al. | 2020 | Saudi Arabia | **** | ** | ** | 8 |
| 183 | Ngoka, et al. | 2020 | Kenya | *** | * | *** | 7 |
| 184 | Paschoal, et al. | 2020 | Brazil | *** | * | *** | 7 |
| 185 | Medkour, et al. | 2020 | Côte d’Ivoire | ** | ** | *** | 7 |
| 186 | Badawi and Yousif. | 2020 | Iraq | ** | ** | *** | 7 |
| 187 | Castro, et al. | 2020 | Brazil | ** | ** | ** | 6 |
| 188 | Cimpan, et al. | 2020 | Romania | **** | * | ** | 7 |
| 189 | Guo, et al. | 2020 | China | *** | ** | ** | 7 |
| 190 | Kaur, et al. | 2020 | India | *** | ** | *** | 8 |
| 191 | Kiouani, et al. | 2020 | Algeria | ** | ** | *** | 7 |
| 192 | Lee, et al. | 2020 | Korea | ** | ** | ** | 6 |
| 193 | Manoj, et al. | 2020 | India | *** | * | *** | 7 |
| 194 | Obeta, et al. | 2020 | Nigeria | *** | ** | *** | 8 |
| 195 | Seleznova, et al. | 2020 | Latvia | *** | ** | *** | 8 |
| 196 | Senthil, et al. | 2020 | India | ** | ** | *** | 7 |
| 197 | Thomas, et al. | 2020 | Colombia | *** | ** | *** | 8 |
| 198 | Wang, et al. | 2020 | China | *** | ** | *** | 8 |
| 199 | Zanet, et al. | 2020 | Italy | ** | ** | *** | 7 |
| 200 | Li, et al. | 2020 | China | ** | ** | ** | 6 |
| 201 | ASLANTAŞ, et al. | 2020 | Turkey | *** | * | *** | 7 |
| 202 | Díaz-Regañón, et al. | 2020 | Nepal | *** | ** | *** | 8 |
| 203 | Habibi, et al. | 2020 | Iran | *** | ** | *** | 8 |
| 204 | Abdullah, et al. | 2021 | Egypt | ** | ** | *** | 7 |
| 205 | Zaki, et al. | 2021 | Egypt | *** | ** | *** | 8 |
| 206 | Bouattour, et al. | 2021 | Tunisia | **** | ** | *** | 9 |
| 207 | Khanmohammadi, et al. | 2021 | Iran | *** | * | ** | 6 |
| 208 | Kopparthi, et al. | 2021 | India | ** | ** | *** | 7 |
| 209 | Preena, et al. | 2021 | India | ** | ** | ** | 6 |
| 210 | Ngoka, et al. | 2021 | Kenya | **** | ** | ** | 8 |
| 211 | Nhuong, et al. | 2021 | Cambodia | *** | * | *** | 7 |
| 212 | García-Quesada, et al. | 2021 | Costa Rica | *** | * | *** | 7 |
| 213 | Zeng, et al. | 2021 | China | ** | ** | *** | 7 |
| 214 | Stayton, et al. | 2021 | U.S.A | ** | ** | *** | 7 |
| 215 | Carli, et al. | 2021 | Italy | *** | ** | *** | 8 |
| 216 | Bawm, et al. | 2021 | Myanmar | **** | ** | *** | 9 |
| 217 | Panda, et al. | 2022 | India | *** | * | ** | 6 |
| 218 | Dordio, et al. | 2022 | Portugal | **** | ** | ** | 8 |
| 219 | Ikejiofor, et al. | 2021 | Nigeria | **** | * | ** | 7 |
| 220 | Morelli, et al. | 2021 | Italy | *** | ** | ** | 7 |
| 221 | Dos Santos, et al | 2022 | Brazil | *** | * | *** | 7 |
| 222 | AbdElmaged. | 2022 | Egypt | ** | ** | *** | 7 |
| 223 | Bordoloi, et al | 2022 | India | ** | ** | *** | 7 |
| 224 | Fonsêca, et al | 2022 | Brazil | *** | ** | *** | 8 |
| 225 | Niestat, et al | 2022 | U.S.A | **** | ** | *** | 9 |
| 226 | Padmaja, et al | 2022 | India | *** | * | ** | 6 |
| 227 | Yang, et al | 2022 | Taiwan | ** | ** | *** | 7 |
| 228 | Aitor, et al. | 2022 | Chile | *** | ** | *** | 8 |
| 229 | Zeng, et al. | 2022 | China | *** | ** | *** | 8 |
